# ERCC6 at the Transcription–Replication Interface: Integration of Transcription-Coupled Repair with Replication Stress Responses

**DOI:** 10.3390/ijms27167396

**Published:** 2026-08-19

**Authors:** Evelyn Zambrano, Fernanda Morales, Yanara A. Bernal, Katherine Marcelain

**Affiliations:** 1 Departamento de Oncología Básico Clínico, Facultad de Medicina, Universidad de Chile, Santiago 8380453, Chile; evelyn.zambrano@ug.uchile.cl (E.Z.); fernmorales@ug.uchile.cl (F.M.); 2 Centro para la Prevención y el Control del Cáncer (CECAN), Facultad de Medicina, Universidad de Chile, Santiago 8380453, Chile; 3 School of Nursing, Pontificia Universidad Católica de Chile, Santiago 8331150, Chile

**Keywords:** replication stress, transcription–replication conflicts, R-loops, ERCC6/CSB, transcription-coupled repair, fork restart, mutagenesis

## Abstract

Replication and transcription share the DNA template and must be coordinated to preserve genome integrity. Although temporally organized across the cell cycle, essential transcriptional programs—encoding replication machinery, canonical histones, and DNA repair factors—operate concurrently with DNA synthesis during S phase. Transcription–replication conflicts (TRCs) therefore constitute a recurrent endogenous source of replication stress (RS), particularly under hypertranscriptional or chromatin-constrained conditions. A frequent outcome of TRCs is the formation of R-loops–RNA:DNA hybrids that can stall or collapse replication forks, leading to DNA damage. This review summarizes current evidence supporting a broader involvement of ERCC6/CSB at the transcription–replication interface beyond its established role in transcription-coupled repair. We discuss how ERCC6 participates in RNA polymerase II processing, chromatin remodeling, R-loop metabolism, replication fork protection, and repair pathway engagement following RS, operating through both its ATPase domain and intrinsically disordered regions. Conversely, in homologous recombination-deficient contexts, *ERCC6* may favor mutagenic restart mechanisms, including break-induced replication; and recent evidence indicates that *ERCC6* status influences cellular fate and the genomic distribution of stress-induced mutations, linking transcription-coupled repair with transcription-dependent mutagenesis. These observations support a model in which ERCC6 coordinates transcription-associated repair with RS responses, shaping genome maintenance and mutational outcomes, with implications for cancer and therapeutic strategies.

## 1. Introduction

The maintenance of genomic integrity is an essential requirement for cellular survival and organismal health [1,2]. Although transcriptional activity is predominantly associated with G1 and DNA replication defines S phase, entry into S phase involves extensive remodeling of the transcriptional landscape rather than a complete shutdown of transcription [3]. Instead, cells actively transcribe genes encoding DNA replication machinery [4], canonical histones needed for chromatin assembly [5], and key factors involved in DNA repair and chromatin remodeling to safeguard replication fork progression [6,7].

DNA replication is inherently challenged by endogenous physical and structural obstacles that can transiently slow or stall replication fork progression [1,2]. Consequently, a basal level of RS is an intrinsic physiological feature of S phase and is generally managed by coordinated RS response pathways, thereby supporting faithful genome duplication and preserving genome stability [1,2,8]. Because DNA replication and transcription share the same DNA template, their simultaneous execution during S phase creates an inherent risk of transcription–replication conflicts (TRCs) [9]. Under physiological conditions, TRCs are generally transient and are controlled by coordinated RS response and DNA repair mechanisms [9]. However, under pathological conditions—including oncogene-driven hypertranscription [10], extensive DNA damage, or defects in genome maintenance mechanisms—the frequency and persistence of TRCs increase, resulting in elevated RS and genome instability [9]. Furthermore, they preferentially accumulate in genomic regions that are intrinsically difficult to replicate, creating “hotspots” of genomic instability [11,12].

Mechanistically, the hazard of TRCs is the formation of R-loops–nucleic acid structures composed of an RNA:DNA hybrid and a displaced single-stranded DNA. Under physiological conditions, R-loops participate in regulatory functions, such as modulating gene expression and organizing chromatin [13,14,15]. However, in the context of TRCs, unscheduled or persistent R-loops act as physical barriers to replication fork progression. These aberrantly stabilized structures can stall the replisome, activate checkpoint signaling, and promote fork collapse, leading to DNA double-strand breaks (DSBs) [16,17,18,19].

Within this framework, ERCC6 (also known as CSB) has been identified as an important functional integrator at the interface between transcription and replication. Traditionally characterized as a core component of transcription-coupled nucleotide excision repair (TC-NER) for removing bulky lesions [20,21], ERCC6 is now also implicated in the regulation of replication fork reversal, fork protection, and restart, particularly under conditions of oncogene-driven or BRCA-deficient RS [22,23]. In this expanded view, ERCC6 operates as a regulatory factor at the transcription–replication interface, a capacity dictated by its activity as an ATP-dependent chromatin remodeling factor and DNA translocase. Unlike general repair factors, ERCC6 selectively recognizes and engages stalled RNA polymerase II (RNAPII), actively remodeling the transcription complex and the surrounding chromatin environment to limit R-loop persistence and facilitate coordinated repair and replication responses [24,25,26].

Current evidence positions ERCC6 not merely as a facilitator of repair, but as a regulatory factor at the transcription–replication interface that influences the fate of stalled replication forks [27]. Its role is context-dependent: ERCC6 contributes to the controlled processing of deleterious, unscheduled R-loops, promoting their conversion into intermediates amenable to repair. In cases of persistent or unresolved R-loops, ERCC6/CSB-dependent processing by the XPG and XPF endonucleases can generate R-loop-associated DSBs that serve as substrates for downstream repair mechanisms [28]. Together, these observations support a model in which ERCC6/CSB couples stalled transcription complexes to downstream XPG/XPF-dependent nucleolytic processing, rather than functioning as a catalytic cleavage factor itself.

Subsequently, ERCC6 modulates the stability and restart of the processed fork. Batenburg et al. have established that ERCC6 is required for replication fork recovery, functioning to limit the engagement of deleterious repair pathways [22,23,29]. Specifically, ERCC6 modulates nuclease access to the replication fork through context-dependent regulatory interactions. By biasing repair pathway choice away from classical Non-Homologous End Joining (NHEJ), it promotes recombination-dependent restart or alternative pathways, such as RAD52-mediated break-induced synthesis [22,29,30]. However, in BRCA1/2-deficient cells, these ERCC6-dependent restart routes may increase reliance on intrinsically error-prone repair mechanisms under conditions of severe or persistent RS. In particular, Break-Induced Replication (BIR) has been associated with elevated mutation rates, genomic rearrangements, and copy-number changes [22,23,31,32]. Thus, ERCC6 acts as a central coordinator, first enabling the nucleolytic processing of transcription–replication obstacles and then channeling the resulting intermediates toward repair pathways. This mechanism balances replication recovery and cell survival against a potential mutagenic cost.

This review focuses on the evolving role of ERCC6/CSB as a central regulatory factor linking transcription-coupled repair to the management of replication forks. We discuss how ERCC6 coordinates the resolution of R-loop-associated TRCs and how this regulatory function influences the mutational outcomes associated with RS, determining whether conflicts are resolved through genome-preserving repair or through mutagenic restart pathways. This highlights the importance of ERCC6 in safeguarding genome stability under transcriptional and RS processes that are major drivers of genomic instability and cancer development.

## 2. Contextual Determinants of Transcription-Replication Conflicts

The probability that a transcription–replication encounter will evolve into a pathological lesion is governed by the identity of the transcription machinery and by structural, topological, and chromatin-associated determinants. Understanding how these features shape the location, orientation, and persistence of TRCs is crucial for defining the regulatory role of ERCC6/CSB.

### 2.1. Polymerase-Specific Transcriptional Environments and ERCC6/CSB Functions

Although ERCC6/CSB has also been implicated in transcriptional processes associated with RNA polymerases I and III, its mechanistic relationship with transcription-coupled repair and TRCs is best established for RNA polymerase II, which therefore constitutes the principal focus of this review. These polymerase-specific functions occur within genomic regions that differ substantially in their transcriptional organization and chromatin context [33]. Pol I transcribes tandemly repeated rDNA units that support extremely high transcription rates and are densely occupied by transcription complexes in a relatively nucleosome-depleted nucleolar environment [34,35,36]. Pol II transcribes protein-coding genes and several classes of non-coding RNAs, often across substantially longer gene bodies, and must continuously overcome nucleosomal barriers during the elongation process [34,35,36]. In contrast, Pol III primarily transcribes short genes encoding tRNAs, 5S rRNA, U6 snRNA, and other small non-coding RNAs, which are frequently located within short nucleosome-depleted DNA regions and terminate at oligo-thymidine tracts [34,35,36]. These differences establish distinct transcriptional environments that may differentially influence the probability and persistence of transcription–replication encounters.

Consistent with these polymerase-specific transcriptional environments, ERCC6/CSB has been associated with all three nuclear transcription systems. In the Pol I system, ERCC6/CSB interacts with Pol I, TFIIH, and XPG, thereby stabilizing the transcription complex and promoting efficient pre-rRNA synthesis. CSB deficiency consequently impairs Pol I recruitment and rRNA synthesis and processing [37,38]. In the Pol II system, ERCC6/CSB acts as a processivity factor, helping Pol II overcome barriers such as nucleosomes or DNA lesions. It recognizes and engages stalled Pol II complexes, promoting TC-NER initiation and transcription recovery [33,39]. In the Pol III system, loss of ERCC6/CSB has been associated with metaphase fragility of the tandemly repeated RN5S locus, which contains Pol III-transcribed 5S rRNA genes, suggesting that CSB contributes to transcriptional elongation or chromatin stability at these loci [40].

Thus, although ERCC6/CSB functions extend across the three nuclear transcription systems, its mechanistic relationship with TC-NER and TRCs is best established for Pol II.

### 2.2. Replication Directionality, Origin Organization, and MCM Licensing Influence Conflict Severity

TRCs differ significantly in severity depending on the relative orientation of the replication fork and the transcription complex. In co-directional encounters, the replisome and transcription machinery move in the same orientation, which generally allows conflict resolution through polymerase bypass or transcription termination. In contrast, head-on collisions occur when the replisome and RNA polymerase II converge. This orientation is associated with drastically increased steric hindrance and impaired replisome progression compared to co-directional encounters [18,19,41,42].

The severity of head-on collisions is not merely physical but topological. As both complexes move toward each other, positive supercoils accumulate in the intervening DNA at a rate that can overwhelm topoisomerases. This “topological crunch” can stall both complexes. Genome-wide analyses and engineered collision systems indicate that orientation is a major determinant of conflict outcome, influencing both fork progression dynamics and the stability of transcription-associated structures. Long and highly transcribed genes are particularly vulnerable to TRCs because their extended transcriptional domains increase the probability of encounters with replication forks [10,43].

Beyond generating conflicts, transcription actively shapes the replication landscape [18,42,44]. Chen et al. demonstrated that replication initiation in human cells is strongly enriched immediately upstream of transcription start sites (TSS), particularly at long genes with high RNAPII occupancy, whereas origin firing is underrepresented within gene bodies [44]. This organization biases replication forks to travel through the 5′ regions of highly transcribed genes in the same direction as transcription, thereby reducing the probability of head-on encounters under unperturbed conditions. Replication termination, in contrast, is enriched at transcription termination sites (TTS) [44]. Under RS, origin activity near active TSSs increases, but initiation also becomes more prominent downstream of genes and replication termination redistributes into gene bodies, increasing the proportion of gene 3′ regions replicated in a head-on orientation [44,45].

This replication landscape is established during G1, when MCM2–7 complexes are loaded onto chromatin in substantial excess over the number of origins ultimately activated during S phase [46,47,48]. The excess pool licenses dormant origins that can support replication completion when forks stall [48,49,50]. At highly transcribed loci, accessible chromatin may favor ORC recruitment, MCM2–7 loading, and subsequent origin activation near TSSs, thereby coupling transcriptional activity to replication initiation [41,50]. In addition, transcriptional and replicative machineries can reposition loaded MCM2–7 double hexamers along DNA, suggesting that ongoing transcription may redistribute licensed origins away from gene bodies and toward adjacent intergenic regions [41,50]. Thus, excess MCM loading provides flexibility under RS, whereas the subset of licensed origins selected for activation determines local fork directionality and the probability of co-directional or head-on encounters [48,50].

Nevertheless, the co-directional organization of replication and transcription cannot prevent conflicts when RNAPII becomes persistently stalled at a DNA lesion [41]. Evidence linking transcriptional arrest to transcription-associated DNA damage is provided by studies using Illudin S, which induces lesions that preferentially block RNA polymerase II on the transcribed strand and are exclusively processed by TC-NER [51]. This illustrates that RNAPII stalling itself constitutes a key trigger for transcription-coupled repair engagement [52]. In head-on encounters, where collision geometry and topological stress already favor polymerase arrest [19], transcription-blocking lesions are therefore expected to be particularly persistent, increasing reliance on ERCC6/CSB-mediated processing [20]. In this context, ERCC6/CSB localizes at stalled RNAPII complexes and coordinates transcription-coupled lesion handling, linking collision orientation, polymerase arrest, and DNA damage responses [42].

### 2.3. Chromatin Architecture and Transcriptional Load

Chromatin organization critically shapes the probability and persistence of TRCs. Increased chromatin compaction, altered histone modifications, and reduced nucleosome mobility restrict access of RNA processing and topological stress-relief factors, thereby limiting efficient conflict resolution [53,54,55,56]. Stress conditions that elevate transcriptional output or alter chromatin fluidity—including deregulated replication timing, histone deacetylase activity, and heterochromatin expansion—are associated with increased conflict persistence and replication fork instability. These chromatin- and transcription-dependent effects establish a structural context in which RS becomes spatially organized rather than stochastic [57,58,59].

In this framework, chromatin state not only dictates where TRCs arise but also how permissive transcription-coupled repair pathways can operate, influencing access to stalled RNAPII and R-loop-prone regions, particularly in regions with reduced accessibility [60,61]. Consistent with this, recent studies utilizing reporter systems for head-on collisions indicate that R-loop-driven TRCs actively drive nucleosome eviction, creating a locally accessible chromatin state. Subsequent recovery of transcription following these conflicts requires specific epigenetic bookmarks, such as DOT1L-mediated H3K79 methylation, suggesting a tightly coordinated cycle of chromatin opening and restoration that is distinct from canonical replication [62,63].

## 3. R-Loops at the Transcription–Replication Interface

A frequent and structurally significant outcome of TRCs is the formation of R-loops. These three-stranded nucleic acid structures consist of an RNA:DNA hybrid and a displaced single-stranded DNA (ssDNA) loop. The metabolism of R-loops represents a critical intersection between transcription regulation and genome stability.

### 3.1. Sources and Structural Basis of R-Loop Formation

R-loops are not intrinsically pathological structures but serve as physiological intermediates in transcription regulation and chromatin organization, and their pathogenicity emerges primarily when mechanisms that restrict their localization and resolution fail [13,15].

R-loop formation is not restricted to transcription–replication collisions and can occur independently of ongoing DNA replication [9,28]. Physiological and pathological R-loops can arise at promoters, transcription termination regions, repetitive loci, and other highly transcribed regions, as well as following defective RNA splicing or processing, impaired RNA export, topoisomerase dysfunction, oxidative DNA damage, or persistent transcriptional pausing [64,65,66,67,68,69]. Replication encounters can subsequently transform these pre-existing structures into particularly harmful barriers, but are not required for their initial formation [19,70].

In this context, their formation is favored by transcriptional pausing, inefficient RNA processing, repetitive or rigid chromatin environments, and unresolved topological stress [16,18]. When R-loops persist, they interfere with replication fork progression, consistent with increased RS and activation of DNA damage responses [15,19].

### 3.2. Collision Geometry and Genomic Features Defining R-Loop Vulnerability

Genome-wide analyses and controlled collision systems further indicate that TRCs represent a major source of pathological R-loop accumulation. Collision geometry modulates hybrid stability and downstream signaling [18,41]. Persistent R-loops expose extended stretches of single-stranded DNA, which may skew the engagement of repair pathways and increase susceptibility to nucleolytic processing at stalled forks [16,17,18,19].

TRCs and R-loops should not be viewed as sporadic replication obstacles but as recurrent structural states that emerge from the architecture of active genomes and require continuous cellular management. In proliferative cells, persistent R-loops tend to be enriched at genomic regions characterized by high transcriptional activity, long gene length, GC-rich promoters, repetitive sequences, and late-replicating heterochromatin. These features converge to create environments with elevated topological stress and impaired hybrid resolution, thereby predisposing replication forks to sustained conflicts [16,18,19].

Mechanistically, collision geometry is a major determinant of whether TRCs stabilize into persistent R-loops. Rather than acting merely as a geometric constraint, orientation defines a landscape of structural vulnerability in which specific genomic regions become predisposed to hybrid persistence, checkpoint engagement, and replication fork instability during the S phase [18,41].

### 3.3. TC-NER as a Regulator of R-Loop Tolerance and Replication Stability

Accumulating evidence indicates that factors classically assigned to transcription-coupled repair also contribute to R-loop homeostasis and to the cellular response to R-loop-associated transcription blocks [71]. Loss of ERCC6/CSB and disruption of TC-NER-associated processing have been linked to persistent RNAPII stalling, increased R-loop accumulation, and heightened transcription-associated genome instability [28]. These observations do not necessarily indicate that TC-NER factors enzymatically dissolve RNA:DNA hybrids; instead, they support a role for transcription-coupled control of RNAPII stalling in limiting R-loop formation and persistence.

ERCC6/CSB acts at two levels of R-loop homeostasis. It exerts a preventive function by promoting RNAPII processivity and limiting prolonged pausing, backtracking, and polymerase drop-off that favor R-loop formation [27,72,73]. It also acts directly at R-loop-associated substrates through its C-terminal RNA:DNA hybrid-binding region [74]. Once recruited, CSB can promote context-dependent downstream processing: its acidic domain interacts with RAD52 and facilitates RAD52 recruitment at ROS-induced R-loops, whereas other persistent pathological R-loops undergo XPG/XPF-mediated cleavage in a CSB-dependent manner [27,28,74]. Consistent with a broader role in R-loop homeostasis, RAD52 also promotes R-loop resolution and limits TRC-associated RS, DNA damage, and mutagenesis [75].

ERCC6/CSB is therefore mechanistically distinct from canonical R-loop-removing enzymes such as RNase H1 and FANCM, but functionally intersects with pathways that determine whether persistent hybrids are unwound, nucleolytically processed, or converted into recombination intermediates. These established and proposed relationships are summarized in Table 1.

Collectively, these pathways indicate that ERCC6/CSB does not function as a canonical R-loop-dissolving enzyme but instead links transcription-coupled control of RNAPII to the context-dependent processing of persistent R-loop-associated structures. Direct biochemical or genetic coordination of ERCC6/CSB with RNase H1 or FANCM has not been demonstrated, supporting the interpretation that these factors operate through mechanistically distinct but potentially complementary pathways of R-loop homeostasis.

### 3.4. Sequence-Encoded Barriers and Human-Specific Vulnerability

Recent work shows that R-loop accumulation in ERCC6-deficient cells is driven by sequence-specific transcriptional barriers rather than global transcriptional dysfunction. Zhang et al. (2024) identified poly-thymidine tracts (“T-runs”) as intrinsic RNAPII pause sites that require ERCC6/CSB for efficient elongation; in its absence, polymerase stalling promotes R-loop formation, particularly poly-thymidine tracts located downstream of G-rich regions, where loss of ERCC6/CSB favors RNAPII drop-off and exposure of the nascent RNA 3′ end, thereby stabilizing RNA:DNA hybrids [72]. Another aspect of major pathological relevance from Zhang et al. (2024) [72] is the evolutionary divergence in the distribution of R-loop-prone sequence motifs. G-rich regions followed by internal T-runs are enriched in long neuronal genes in the human genome but are markedly less prevalent in mice. This difference provides a mechanistic explanation for why Ercc6-deficient mice develop cancer yet fail to recapitulate the severe neurodegenerative and progeroid phenotypes of Cockayne Syndrome (CS), reflecting a higher dependence of the human neuronal genome on ERCC6/CSB to overcome sequence-encoded transcriptional barriers [72].

Together, these findings position R-loops as transcription-associated structural intermediates whose biological consequences are determined by their sequence and genomic context, persistence, cell-cycle stage, and mode of processing. ERCC6/CSB acts as a molecular coordinator at this interface by promoting RNAPII processivity, limiting pathological R-loop stabilization, and influencing the context-dependent processing of persistent structures. When these lesions persist and are subsequently encountered by the replisome, they can trigger checkpoint activation, fork remodeling, or nucleolytic processing. Depending on lesion burden and repair outcome, cells may undergo cell-cycle arrest, activate apoptosis, or survive through repair and restart mechanisms that can impose a mutagenic cost [79,80]. The evolutionary enrichment of sequence-encoded transcriptional barriers in long human neuronal genes provides an organism-level link between these molecular and cellular outcomes and the neurodegenerative and progeroid manifestations of Cockayne syndrome.

## 4. ERCC6/CSB as a Multifunctional Integrator in Transcription–Replication Conflicts

While TC-NER supports a conceptual framework for R-loop surveillance (Section 3), ERCC6/CSB emerges as a multifunctional integrator of the molecular processes operating at TRCs. ERCC6/CSB contributes to lesion recognition, RNA polymerase II handling, chromatin remodeling, and RS responses, thereby facilitating the stabilization, processing, or removal of stalled transcription complexes during ongoing DNA replication [20,24,26,81].

Furthermore, the biological outcome of TRCs is highly context-dependent. Multiple factors influence whether stalled replication forks are efficiently restarted or instead progress toward genome instability, including the DNA polymerases engaged during repair synthesis, the choice of DSB repair pathway, the genomic context of the lesion, the transcriptional burden, and the intensity and duration of RS [1,10,29,41,80]. Rather than acting in isolation, ERCC6 functions within this network to coordinate transcription recovery while promoting replication fork stability and genome integrity [26,82,83].

**Figure 1:**
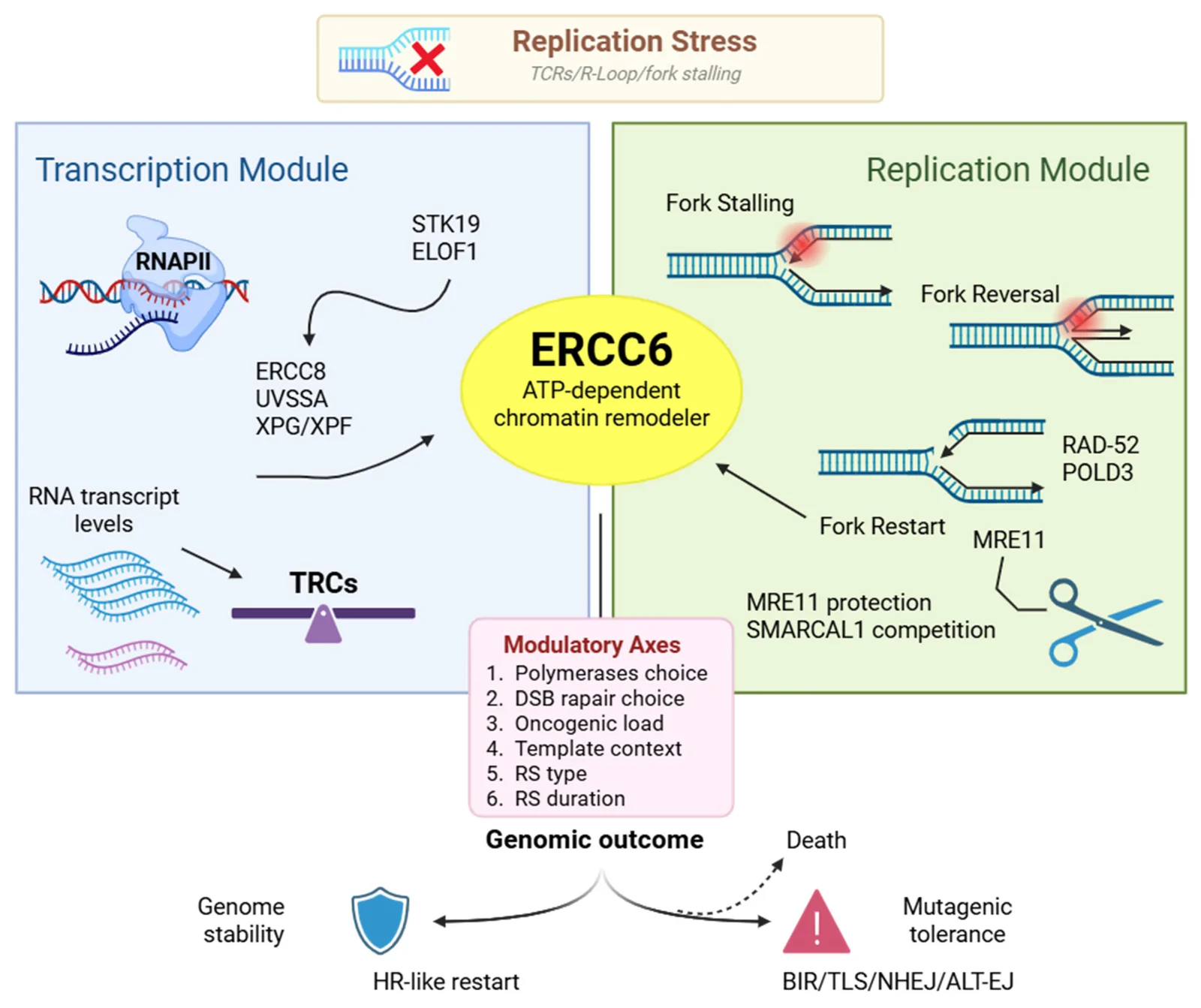
ERCC6/CSB integrates transcription-coupled repair with replication stress responses at transcription–replication conflicts. Schematic model illustrating ERCC6/CSB as an ATP-dependent chromatin remodeler positioned at the interface between transcription and DNA replication. RS arising from TRCs, R-loops, or fork stalling creates structural challenges that engage coordinated responses in both transcriptional and replication programs. On the transcriptional side (left), elongating RNAPII, transcriptional pausing, and increased transcriptional output (RNA transcript levels) elevate the likelihood of encounters between transcription and replication machinery, promoting TRCs and activation of TC-NER pathways involving STK19, ELOF1, ERCC8, UVSSA, and the XPG/XPF endonucleases [20,84,85,86]. On the replication side (right), RS promotes fork stalling, fork reversal, and restart processes involving factors such as RAD52, POLD3, and the MRE11 complex [22,25]. Positioned between these modules, ERCC6 influences chromatin accessibility, RNAPII processing, and replication fork dynamics. The outcome of these coordinated responses is further shaped by contextual determinants, including the choice of DNA polymerase (e.g., translesion synthesis (TLS) polymerases), the DSB repair choice (HR vs. NHEJ), the overall oncogenic load, the genomic template context (e.g., repetitive sequences, levels of the transcript), the type of RS (e.g., inducing agent (Hydroxyurea, Camptothecin), and its duration (acute or chronic). These integrated responses ultimately determine genomic outcomes, ranging from genome stability through high-fidelity mechanisms such as HR to mutagenic tolerance through error-prone repair or restart pathways. However, when damage exceeds repair capacity, cell death programs are activated.

A schematic overview of these interconnected molecular pathways and the proposed role of ERCC6 at transcription–replication conflicts is shown in Figure 1.

### 4.1. ERCC6/CSB: Integrating TC-NER with Replication Fork Fate Decisions

CSA, UVSSA, and ELOF1 coordinate RNAPII ubiquitylation or inactivation and promote TFIIH recruitment, while STK19 stabilizes this repair platform by stimulating RNAPII ubiquitylation and facilitating productive recruitment of UVSSA and TFIIH [20,84,85,86]. In addition, ERCC6/CSB interfaces with transcription elongation machinery through its interaction with LEO1, a component of the PAF1 complex, and both factors are co-recruited to transcription-blocking lesions. Loss of LEO1 phenocopies ERCC6/CSB deficiency in cellular sensitivity to genotoxic stress, supporting a functional link between RNAPII elongation control and transcription-coupled DNA repair [83].

At sites of TRCs, TC-NER contributes to the processing of transcription-associated DNA lesions and participates in R-loop handling [28]. In parallel, ERCC6–PARP1/2 regulate single-strand break repair in actively transcribed regions and modulate ERCC6/CSB association with chromatin under oxidative stress [87,88,89]. Consistently, ERCC6/CSB also intersects with base excision repair pathways and participates in the recognition and processing of oxidative DNA lesions that recruit TC-NER components [90].

Beyond RNAPII-associated lesions, ERCC6/CSB also plays a direct role in replication-associated DNA damage tolerance. While initially described at oxidative damage sites and telomeres, recent proteomic and genomic analyses reveal a broader interaction between ERCC6 and RAD52 [25]. RAD52 has been shown to interact directly with RNA Polymerase II and recruit Topoisomerase IIα (TOP2A) to R-loop-associated sites [75]. In this model, ERCC6 may promote an RNAPII-associated environment permissive to RAD52–TOP2A activity to alleviate the topological stress (supercoiling) characteristic of head-on collisions, thereby preventing fork collapse before nucleolytic processing becomes necessary [25,75]. Thus, the combined evidence suggests that ERCC6/CSB, RAD52, and TOP2A may functionally converge at a subset of conflict-prone loci to decouple transcription-associated supercoiling from irreversible fork failure.

### 4.2. Decision Control over RNAPII at Persistent Transcription Blocks

In addition to coordinating repair and replication-associated processes, ERCC6/CSB also governs the fate of RNA polymerase II at sites of persistent transcriptional arrest. Recent work indicates that when TC-NER is compromised, stalled RNAPII is cleared through a p97 (VCP)- and proteasome-dependent pathway, providing an alternative mechanism for removing irreversibly blocked transcription complexes [91,92].

In TC-NER-proficient cells, lesion-stalled RNAPII is predominantly resolved through TC-NER-mediated lesion removal, allowing transcription to resume, whereas in UVSSA-deficient cells, CRL4CSA- and VCP/proteasome-dependent degradation of stalled RNAPII becomes the dominant route to clear transcription-blocking lesions at the expense of aborting transcription [92]. This hierarchy is enforced by CSA-mediated ubiquitination of RNAPII and by UVSSA-dependent recruitment of the deubiquitinase USP7, which promotes efficient assembly of the TC-NER machinery and stabilizes ERCC6/CSB, thereby favoring repair over eviction rather than directly inhibiting the p97 pathway [91].

Beyond ubiquitination-driven fate decisions, additional regulatory layers modulate how efficiently ERCC6/CSB engages lesion-stalled RNAPII and commits complexes to productive repair. Complementary regulatory input arises from the ARK2N–CK2 complex, which phosphorylates ERCC6/CSB and licenses stable engagement with lesion-stalled RNAPII. Disruption of this signaling axis impairs efficient TC-NER initiation and may shift the balance between productive repair and proteasome-mediated removal of stalled polymerase in the experimental systems analyzed [93]. However, independent work has reported that transcription-coupled DNA repair can proceed normally in ARK2N-deficient cells, arguing that ARK2N is not a universal core component of the TC-NER machinery [94].

### 4.3. ERCC6/CSB Engagement with Non-Canonical DNA Structures at Transcription–Replication Conflicts

The functions of ERCC6/CSB at TRCs are not limited to protein complexes stalled at DNA lesions. ERCC6/CSB also engages non-canonical DNA structures that can obstruct both transcription and replication, thereby directly challenging transcription–replication coordination. Among the diverse endogenous obstacles posed by genome architecture, DNA secondary structures constitute a major class of transcription–replication barriers [54]. In particular, G-quadruplex (G4) DNA structures represent significant impediments to transcription and replication, with recent work revealing a selective role of ERCC6/CSB in resolving intermolecular G-quadruplexes (iG4s) rather than canonical intramolecular G4s [95]. ERCC6/CSB binds iG4s with picomolar affinity, particularly within ribosomal DNA arrays, and exhibits a non-ATP-dependent destabilizing activity, supporting a specialized role in nucleolar function and genome architecture. This dual mode of engagement is consistent with a modular structural organization, in which flexible, intrinsically disordered regions may facilitate high-affinity electrostatic interactions with complex DNA topologies, while the ATPase motor domain provides the mechanical force required for remodeling and resolution of more stable chromatin-associated barriers [26,96].

Consistently, ERCC6/CSB-deficient cells accumulate G4 structures in the nucleolus and display transcriptional stalling and rRNA processing defects, hallmarks of CSB pathology [95]. Collectively, these findings redefine iG4s as long-range architectural elements rather than local DNA features and position ERCC6/CSB as a context-dependent chromatin-associated factor operating within higher-order genome organization, particularly in transcriptionally active repetitive domains. Emerging models further suggest that multimolecular G4 assemblies contribute to transcriptional compartmentalization and chromatin organization [97,98]. Within this broader framework, ERCC6/CSB may operate in a dynamic landscape in which DNA topology and chromatin state influence the spatial distribution and resolution of TRCs [26,95].

## 5. Repair Versus Mutagenesis: How the Resolution of TRCs Shapes Genomic Outcomes

RS spans a spectrum from fork slowing and uncoupling to fork reversal and, ultimately, fork collapse and DSB formation. When stalling persists, forks undergo structural remodeling, including reversal, and may become substrates for structure-specific nucleases; in the absence of adequate protection or restart mechanisms, stalled forks can progress toward DSB formation [1,32,99,100].

When ATR–CHK1 checkpoint signaling remains intact, replication fork stabilization, suppression of inappropriate origin firing, and preservation of fork integrity favor homology-directed repair outcomes, and HR is generally considered to restore replication continuity with relatively high fidelity [1,25,101,102,103]. In contrast, nucleolytic processing followed by RAD52-dependent break-induced replication (BIR) or engagement of error-prone DNA synthesis is associated with increased mutational burden and structural variation [23,32].

When stalled forks collapse, MUS81-dependent cleavage can enable replication restart but generates double-strand breaks (DSBs) that increase the risk of genome rearrangements [99,100]. ERCC6/CSB has been functionally linked to RAD52-dependent fork restart and BIR-like repair pathways at collapsed forks, reinforcing its role in error-prone replication recovery programs [104]. In HR-deficient contexts, this shift appears particularly pronounced, as repair depends more strongly on compensatory mechanisms with higher mutational potential [23,32]. Similar principles apply at specialized genomic regions: at ROS-damaged telomeres, ERCC6/CSB helps channel telomeric R-loops into RAD52–POLD3-dependent BIR-like restart, a process that maintains telomere integrity but may also favor repeat copying and local copy-number change [25].

In mammalian cells, ERCC6/CSB directly influences repair outcome at collapsed or persistently stalled forks by modulating both nucleolytic processing and restart pathway choice. In BRCA1/2-deficient contexts, under conditions of severe or persistent Top1-induced RS, ERCC6/CSB promotes MRE11-driven resection and coordinates RAD52-dependent break-induced replication following MUS81-mediated cleavage, thereby coupling fork restart to pathways associated with elevated mutational risk [22,23,29]. Mechanistically, ERCC6/CSB binds the RPA32 subunit via a conserved RQK (R176/Q177/K178) motif and directly competes with SMARCAL1 for access to RPA-coated stalled forks. In BRCA2-deficient cells, this interaction promotes fork slowing and MRE11-dependent degradation, whereas SMARCAL1 counteracts BIR-like restart and limits genome instability [22]. This activity is tightly regulated in cells and depends on cell-cycle cues, as ERCC6/CSB–MRE11 engagement at stalled forks has been shown to require CDK-dependent phosphorylation of ERCC6/CSB, restricting fork degradation to pathological contexts such as BRCA1/2 deficiency [82].

Checkpoint and chromatin-associated signals further modulate ERCC6/CSB function; in particular, ATM and CDK2 regulate ERCC6/CSB phosphorylation interaction with RIF1, influencing the HR vs. NHEJ choice at DSBs, providing an additional layer by which RS responses can impact long-term genomic outcomes [30]. In parallel, sequestration of RPA at persistently stalled forks can inhibit nucleotide excision repair in trans, indirectly shifting repair toward alternative, potentially less faithful mechanisms [105]. This integrative view of ERCC6/CSB as a gatekeeper of RS responses has been explicitly developed in recent reviews, which position ERCC6/CSB as a coordinator of transcription, repair, and fork stability with potential relevance in cancer biology [82].

Several axes influence whether TRCs resolution favors genome maintenance or mutagenesis:

1. *Error-prone polymerase deployment:* Under RS or persistent TRCs, repair-associated DNA synthesis may shift from high-fidelity replicative polymerases (Pol δ/ε) toward translesion synthesis (TLS) polymerases such as Pol κ, increasing the risk of base substitutions and small indels [106,107,108]. Biochemical and genomic studies show that Pol κ efficiently bypasses bulky lesions but is intrinsically error-prone on undamaged templates, while Pol δ can promote chromosomal rearrangements and imprecise DSB repair when engaged outside its canonical replicative context [106,107]. In the context of nucleotide excision repair, including TC-NER, gap-filling synthesis can be executed by multiple polymerases depending on cell-cycle stage and chromatin context; however, no single dedicated polymerase has been conclusively established as specific for TC-NER. Current models indicate that polymerase choice is flexible and condition-dependent, raising the possibility that engagement of TLS polymerases during excision-associated resynthesis may contribute to mutational heterogeneity in transcriptionally active regions [109].
2. *DSB repair pathway choice:* Chromatin state, 53BP1–RIF1 signaling, H2AX-dependent chromatin remodeling, and associated factors (including ERCC6) influence the balance between HR and non-homologous end joining (NHEJ) [30,110,111,112]. At collapsed forks, NHEJ is frequently linked to elevated rearrangement risk [113], whereas HR- or BIR-like solutions differ in their propensity to generate copy-number alterations [114].
3. *Transcriptional and oncogenic load:* Hypertranscription increases TRCs and can deplete limiting resources such as dNTP pools, sensitizing cells to replication-associated mutagenesis and promoting reliance on error-prone tolerance pathways [10,43,115,116,117].
4. *Template context:* Repetitive DNA, secondary structures, and heterochromatin environments predispose to stalling, reversal, and breakage, thereby shaping repair pathway engagement [53,54,55,56,59].

**Figure 2:**
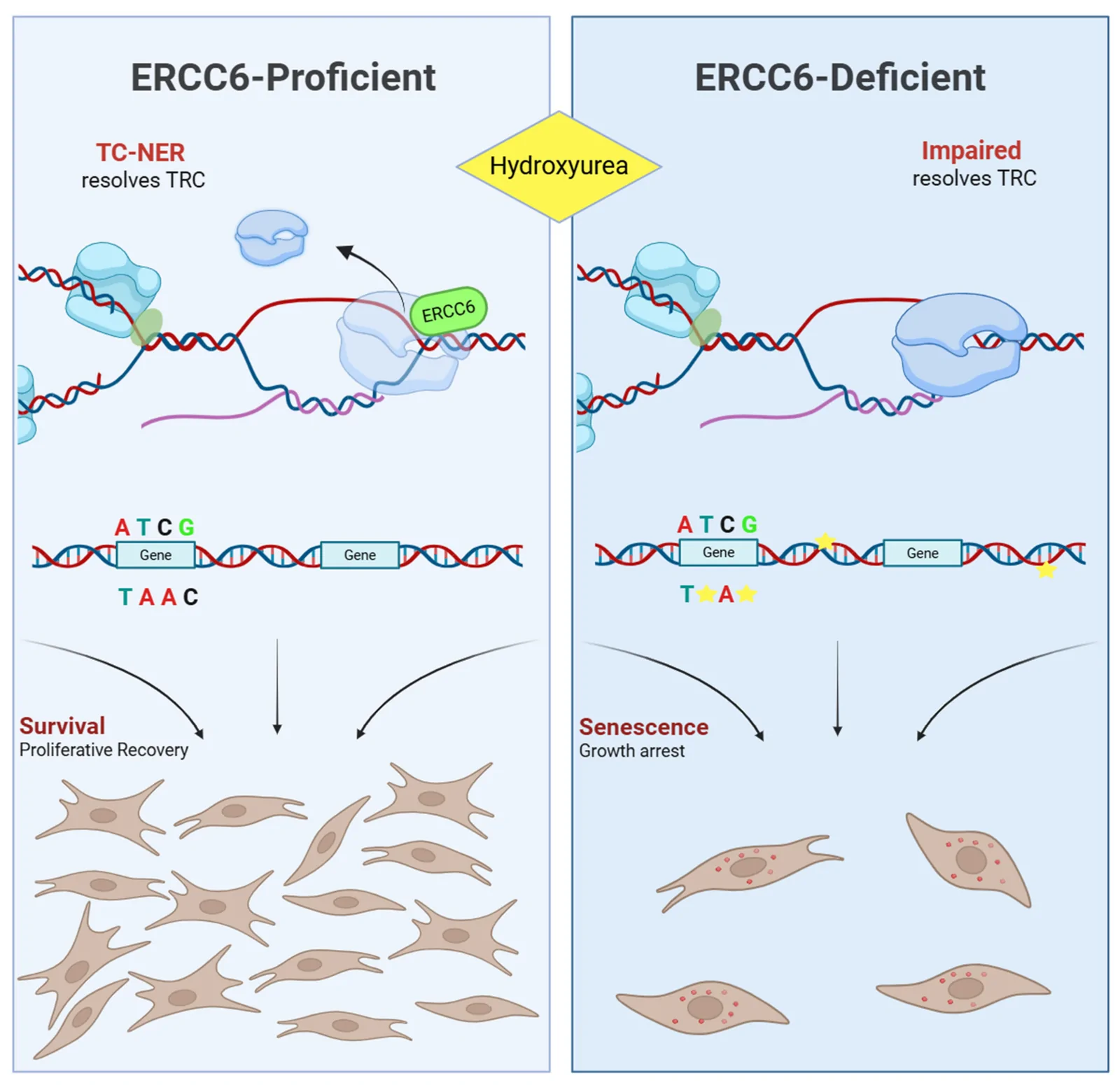
ERCC6 modulates transcriptional responses and genome stability under RS. Comparative schematic of the cellular response to hydroxyurea-induced RS in ERCC6-proficient and ERCC6-deficient cells. In ERCC6-proficient cells under RS, ERCC6 is recruited to sites of TRCs, facilitating the resolution of stalled replication forks and promoting replication recovery. This supports continued cellular proliferation and is associated with the accumulation of mutations, particularly within gene-associated regions. In contrast, in the absence of ERCC6, TRCs persist, leading to RS-induced DNA damage that is not efficiently resolved. This results in the accumulation of DNA lesions (yellow stars) and a cellular phenotype characterized by proliferative arrest and senescence. Over time, cells may process these lesions through alternative repair pathways, potentially generating sequence alterations, while unrepaired damage may also persist in non-proliferating cells due to limited engagement of replication-coupled repair mechanisms.

Support for the mutagenic potential of TRCs also comes from bacterial systems. In bacteria, the transcription–repair coupling factor Mfd (a functional analogue of ERCC6/CSB) biases repair toward error-prone pathways at conflicts, illustrating how transcription-coupled processing can promote adaptive mutagenesis under stress [118,119,120,121]. Evidence from mammalian systems suggests that ERCC6 may exert a comparable context-dependent influence on mutational outcomes during RS. Recent integrative analyses combining imaging, transcriptomic, and whole-exome sequencing approaches suggest that ERCC6 status shapes both cellular fate and the genomic distribution of stress-induced mutations (Figure 2). In human fibroblasts exposed to RS, ERCC6-deficient cells exhibit impaired resolution of TRCs, early engagement of 53BP1-dependent repair pathways, and progressive senescence. In contrast, ERCC6-proficient cells maintain transcriptional continuity and replication restart, allowing proliferative recovery but at the cost of retaining stress-associated mutations preferentially within the coding regions of transcriptionally active loci [80].

These findings suggest that ERCC6 does not simply suppress genome instability but instead modulates how replication-associated lesions are resolved across the genome, influencing whether damage is eliminated through proliferative arrest or retained within the surviving cell population. In this context, ERCC6-dependent processing of transcription-associated lesions may contribute to shaping mutational landscapes under sustained RS.

## 6. Implications in Cancer and Therapeutic Perspectives

Most human pathologies associated with TRCs arise from deregulation of transcriptional output, RNA processing, or R-loop metabolism. In contrast, germline defects in core replication factors are comparatively rare and often incompatible with cellular viability, consistent with transcriptional dysregulation as a major source of TRC-related disease [45]. In this context, ERCC6 functions as a critical modulator of genome stability.

### 6.1. Replication Stress as a Therapeutic Vulnerability in Cancer

Mounting evidence supports that RS is not only a hallmark of many tumors, but also a therapeutic vulnerability that can be exploited by targeting RS adaptation pathways [122,123,124]. In cancer, RS is exacerbated by oncogene activation, transcriptional reprogramming, and epigenetic dysregulation [1,8,125,126]. Consistent with this, global increases in transcriptional activity have been identified as a major contributor to RS in tumor cells [10,43].

The clinical impact of ERCC6 is traditionally defined by loss-of-function mutations leading to CS, characterized by extreme sun sensitivity and neurodegeneration. However, a distinct paradigm emerges in oncology: rather than loss of function, ERCC6 overexpression is frequently observed in multiple cancer types. This upregulation provides a selective advantage to tumor cells by enhancing DNA repair capacity, conferring apoptotic resistance, and increasing proliferative potential under conditions of chronic RS [127,128].

Furthermore, the role of ERCC6 in disease extends beyond pathogenic mutations to include population-associated genetic variants that may modulate cancer susceptibility. A recent example is the M1097V variant, which has been reported at a higher frequency among African American patients in a small Louisiana prostate cancer cohort [129]. Unlike CS-associated loss-of-function mutations, M1097V does not abrogate ERCC6 activity but instead enhances the repair of UV-induced cyclobutane pyrimidine dimers and increases cellular tolerance to UV damage [130]. Together, these findings suggest that subtle changes in ERCC6 activity may influence tumor adaptation and treatment response.

### 6.2. Mechanistic Drivers of Vulnerability: From ecDNA-Associated Stress to RNA Processing

**Figure 3:**
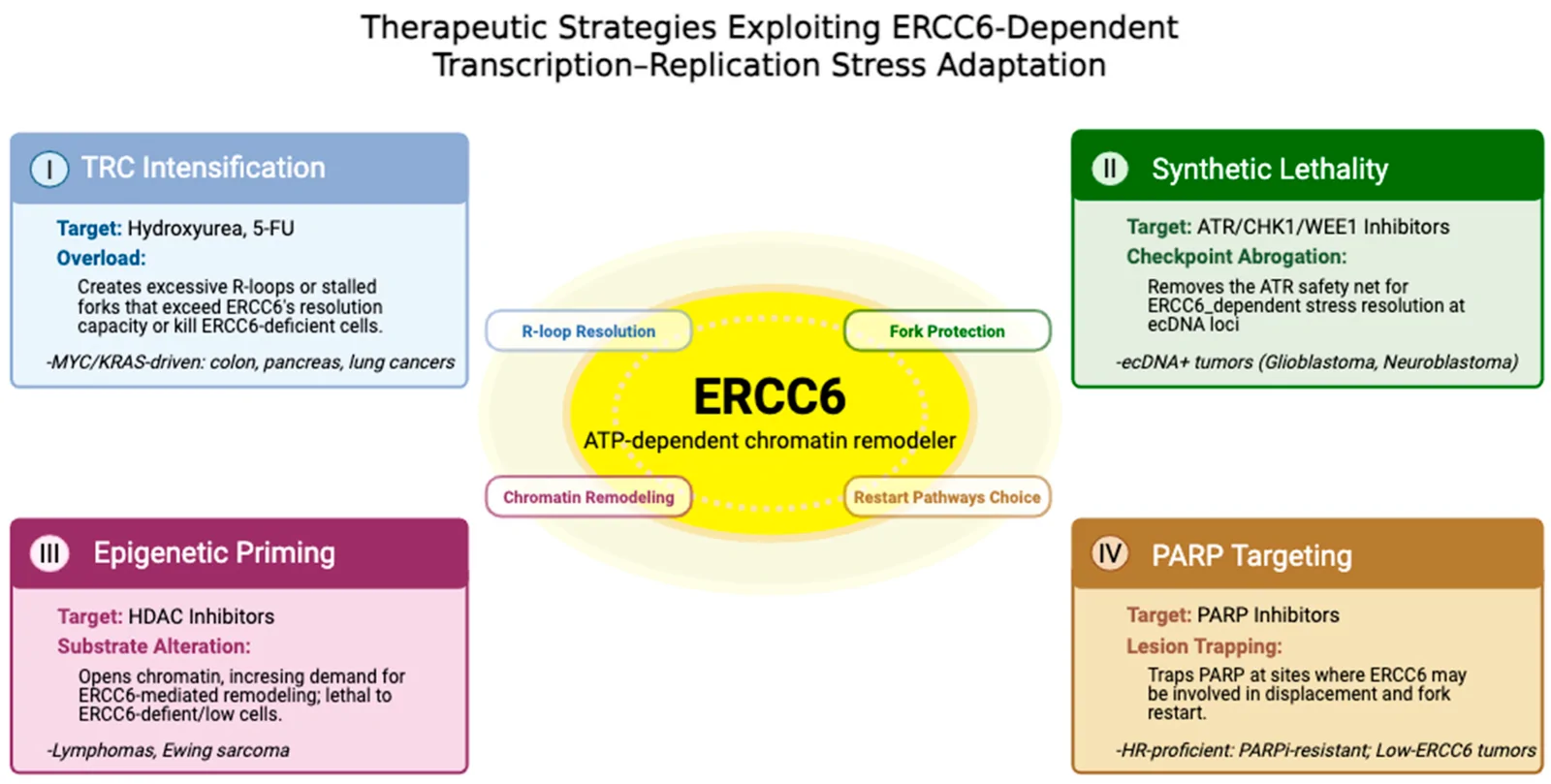
Therapeutic Strategies Exploiting ERCC6-Dependent Transcription–Replication Stress Adaptation. Schematic representation of ERCC6/CSB as an ATP-dependent chromatin remodeler coordinating the resolution of TRCs. ERCC6 contributes to R-loop resolution, chromatin remodeling, replication fork protection, and restart pathway choice, thereby facilitating the recovery of stalled replication forks and maintaining genome stability. Under conditions of elevated transcription-associated RS, ERCC6-dependent pathways influence cellular responses that may be therapeutically exploited. Four conceptual strategies are illustrated: (I) TRCs intensification, where agents such as hydroxyurea or 5-fluorouracil increase RS beyond the buffering capacity of ERCC6-deficient cells; (II) synthetic lethality, in which ATR/CHK1/WEE1 inhibition disrupts checkpoint-mediated stress resolution; (III) epigenetic priming, where HDAC inhibitors increase chromatin accessibility and transcriptional load; and (IV) PARP targeting, where PARP inhibition traps repair intermediates at sites involving ERCC6-dependent fork restart. Together, these mechanisms highlight potential vulnerabilities associated with ERCC6-mediated RS responses in cancer.

The therapeutic potential of exploiting ERCC6-dependent stress adaptation pathways stems from its multifaceted role in managing molecular stressors that are pathologically elevated in cancer cells (Figure 3). These vulnerabilities arise not only from classical defects in DNA repair but also from the unique structural, transcriptional, and regulatory features of the cancer genome.

#### 6.2.1. ecDNA and R-Loop-Induced Replication Stress

Emerging evidence supports a link between R-loops and the initiation of intra- and extrachromosomal DNA amplification, including ecDNA formation, thereby directly linking transcription-associated structures to oncogene copy-number gains and therapy resistance in cancer [131]. Once established, ecDNAs adopt a hyperaccessible chromatin topology that sustains extreme transcriptional output, which is associated with pervasive accumulation of R-loops and constitutive TRCs.

This chronic RS renders ecDNA-containing tumors hypersensitive to S-phase checkpoint inhibition, particularly CHK1 and WEE1 inhibitors, which drive these cells into replication catastrophe and apoptosis [132,133]. Thus, the same transcriptional advantage that underlies oncogene amplification on ecDNAs creates synthetic lethality through excessive reliance on conflict-resolution pathways. Although direct mechanistic evidence is still emerging, it is plausible that TC-NER factors such as ERCC6/CSB modulate this process by mediating the resolution of R-loops and preventing their conversion into repairable DSBs via aberrant pathways.

#### 6.2.2. Non-Canonical Roles in RNA Processing and Splicing

Beyond its structural functions at sites of transcription–replication interference, ERCC6 exerts tumor-specific effects by modulating RNA metabolism, a non-canonical activity that can directly shape therapeutic response. This is exemplified in osteosarcoma, where ERCC6 has been implicated in chemotherapy resistance through regulation of alternative splicing rather than direct DNA repair [134].

Studies using patient-derived organoids and functional genetic screens identified ERCC6 as a determinant of cisplatin resistance via its interaction with the splicing regulator HNRNPM. Specifically, elevated ERCC6 expression promotes exon 2 skipping in the pro-apoptotic gene BAX, introducing a premature stop codon and generating a truncated, non-functional isoform that abrogates BAX-mediated apoptosis. Conversely, ERCC6 depletion restores pro-apoptotic BAX splicing and resensitizes tumor cells to cisplatin-induced cell death [134]. Together, these findings uncover a previously unrecognized axis of ERCC6-mediated therapy resistance and highlight its role as a key integrator of cellular stress responses at both the DNA and RNA levels.

### 6.3. Exploiting the ERCC6–Replication Stress Axis for Cancer Therapy

The intrinsic reliance of tumors with high transcriptional burden on ERCC6-dependent stress adaptation pathways provides a potential opportunity for therapeutic intervention. By combining agents that exacerbate RS with inhibitors of stress-signaling and checkpoint pathways, cancer cells can be driven beyond their tolerance threshold, resulting in replication catastrophe and loss of viability.

#### 6.3.1. Intensifying Transcription–Replication Conflicts

One of the most promising translational implications of ERCC6 biology lies in its integration within synthetic lethal frameworks. Genetic and pharmacological evidence supports the idea that the efficacy of RS-inducing therapies depends on a tumor’s capacity to stabilize and restart stalled replication forks under sustained stress [1,2,122].

Pharmacological strategies that intensify TRCs, including agents that increase transcriptional output, restrict dNTP availability (e.g., hydroxyurea, 5-fluorouracil), or induce replication-associated DNA lesions, are increasingly designed to exploit the intrinsic vulnerability of cancer cells to RS [10,43,97,122,123,135]. This vulnerability is further amplified in oncogene-driven settings in which transcription and S-phase entry are simultaneously enhanced.

Oncogenic mutations in RAS and transcriptional deregulation driven by MYC are among the most frequent and clinically significant alterations in several common solid tumors, particularly pancreatic ductal adenocarcinoma (PDAC), colorectal cancer (CRC), and lung adenocarcinoma (LUAD) [136,137,138,139,140]. As an emergent consequence of sustained oncogenic signaling, oncogenic drivers such as MYC and RAS substantially elevate transcriptional output and perturb replication dynamics, which can promote replication fork stalling, TRCs, and genomic instability [43,141,142,143]. MYC overexpression amplifies RNA synthesis and increases replicative demand, heightening TRCs incidence and fork stalling. Similarly, RAS pathway activation has been associated with replication defects and endogenous RS, contributing to genomic instability and tumor progression.

Consistent with this model, PDAC provides functional evidence that TRCs constitute a major, druggable source of endogenous DNA damage in oncogene-driven settings [144]. Using RNAPII–PCNA PLA as a TRCs readout, the authors show that KRAS-driven PDAC cells display high basal TRCs and that pharmacologic trapping of TRCs with the PCNA inhibitor AOH1996 increases RNAPII–PCNA proximity and induces DNA damage in a manner that is at least partially transcription-dependent (attenuated by transcription inhibition), supporting TRCs—not only RS per se—as a mechanistic driver of genome instability and a therapeutic vulnerability in PDAC [144].

#### 6.3.2. Synthetic Lethality via Replication Stress and DNA Repair Dependencies

In this context, combinations with PARP inhibitors and checkpoint pathway inhibitors (e.g., ATR, CHK1, WEE1) have demonstrated synergistic antitumor activity, although therapeutic outcomes depend on how efficiently tumors stabilize stalled replication forks and reinitiate DNA synthesis under stress conditions [135,145,146,147,148,149,150]. Given its emerging role in replication fork restart and processing, ERCC6/CSB may influence whether such stress is resolved through survival-promoting repair mechanisms or through tolerance pathways associated with elevated mutational risk, particularly in homologous recombination-deficient settings [22,23,29].

#### 6.3.3. ecDNA-Driven Replication Stress as a Synthetic Lethal Vulnerability

Furthermore, as outlined in Section 6.2.1, ecDNA amplifications generate persistent TRCs and elevated RS due to sustained transcriptional activity and R-loop accumulation. This pre-existing vulnerability provides a rationale for synthetic lethal approaches based on S-phase checkpoint inhibition, whereby disruption of ATR–CHK1–WEE1 signaling exacerbates RS beyond the tolerance threshold of ecDNA-containing tumors. EcDNA is particularly common in glioblastoma, where oncogene-driven ecDNA correlates with poor prognosis and intratumoral heterogeneity, and contributes to disease aggressiveness [151]. In neuroblastoma, ecDNA also drives oncogene expression and cellular heterogeneity, suggesting potential targetable dependencies linked to RS and checkpoint control [152].

#### 6.3.4. Chromatin-Modifying Therapies and Replication Stress

Epigenetic therapies further intersect with this axis. Histone deacetylase inhibition alters chromatin accessibility and replication dynamics, thereby exacerbating RS and sensitizing tumor cells to DNA damage-based therapies [57,58]. Rather than acting as direct genotoxic agents, HDAC inhibitors impose a substrate-level perturbation that destabilizes the balance at the transcription–replication interface. Under such conditions, cells are expected to rely more heavily on adaptive mechanisms that coordinate chromatin remodeling with transcription-coupled stress responses, providing a conceptual framework in which TC-NER-associated factors, including ERCC6, may become functionally relevant.

In cancer cells, HDAC inhibitors have broad antitumor activity that goes beyond transcriptional reprogramming. These agents have been shown to disrupt DNA replication and the cellular response to RS by downregulating key replication factors such as RRM1, RRM2, CHK1, WEE1, and components of the prereplication complex, thereby impairing replication and increasing RS in tumor models such as Ewing sarcoma cells [153]. In this context, the heightened RS and destabilization of chromatin structure sensitize cancer cells to additional stressors, including DNA-damaging agents and replication checkpoint inhibitors. Moreover, HDAC inhibition has been reported to impair DNA repair pathways and sensitize cancer cells to ionizing radiation and DNA-damage-based therapies by compromising homologous recombination and other repair processes [154]. These functional effects in diverse cancer models provide a rationale for why chromatin substrate alteration driven by HDAC inhibitors could increase reliance on chromatin remodeling and conflict-resolution factors such as ERCC6.

#### 6.3.5. Emerging Strategies for ERCC6/CSB Modulation

Despite growing evidence supporting ERCC6/CSB as a determinant of tumor stress adaptation and therapeutic resistance, direct pharmacological targeting of ERCC6 remains largely unexplored, and no selective ERCC6 inhibitor has yet been developed. A major challenge is its highly conserved ATPase/translocase domain, which may limit the selectivity of conventional ATP-competitive inhibitors [82,155]. Alternative approaches may therefore include transcriptional depletion of ERCC6 or disruption of non-catalytic protein–protein interactions [96,156]. Indeed, gene-silencing strategies already provide preclinical proof of concept that ERCC6 depletion can enhance sensitivity to genotoxic therapies [82,96,134,157].

In this scenario, targeted protein degradation (TPD) may offer a highly promising future strategy. Unlike catalytic inhibitors, which only block its translocase activity, PROTACs or molecular glues could physically eliminate ERCC6. This would simultaneously ablate both its ATP-dependent remodeling and its non-catalytic interaction/scaffolding functions, such as the recruitment of essential NER factors and histone chaperones [39,158]. Such degrader-based strategies could ultimately complement platinum-based chemotherapy, PARP inhibition, or replication-checkpoint targeting in tumors that depend on ERCC6-mediated tolerance to transcriptional and replication stress. In addition, ERCC6 depletion could potentially limit the retention of stress-induced mutations in highly transcribed coding regions, thereby constraining somatic diversification and tumor evolution [80].

### 6.4. Beyond Oncology: ERCC6 and Innate Immune Signaling

Beyond oncology, RS arising at the transcription–replication interface may also contribute to inflammatory signaling in non-cancer contexts. Diverse pathogens can perturb host DNA metabolism in ways that resemble endogenous RS, including depletion of RPA pools, disruption of replication–repair coordination, and replication fork instability [159,160,161,162,163]. These perturbations recapitulate key features of TRCs, which are frequently associated with transcriptional arrest and R-loop accumulation. Although direct mechanistic evidence remains limited, current observations suggest that factors linking transcription-coupled repair with RS responses—such as ERCC6/CSB—could contribute to buffering transcription-associated RS across diverse contexts. Persistent R-loops and genome instability have also been linked to micronucleus formation and activation of cytosolic DNA-sensing pathways, including cGAS-dependent inflammatory signaling [164,165,166]. In line with this concept, recent work by Cancado et al. (2025) has identified a noncanonical cGAS–STING pathway capable of driving cellular and organismal aging independently of acute infection [167], suggesting that inflammatory signaling may emerge as a downstream consequence of persistent genome instability. Although this mechanism has not been directly demonstrated in CS, evidence suggests that ERCC6 deficiency—through its impact on transcription stress, replication dynamics, and R-loop homeostasis—could indirectly influence inflammatory signaling as a secondary outcome of transcription-associated genome instability [164].

## 7. Concluding Remarks

RS emerges at the transcription–replication interface when R-loops and transcriptional barriers stall replisomes, shifting cells from checkpoint-stabilized slowing to fork collapse and recombination-dependent restart if conflicts persist. Within this nexus, ERCC6/CSB functions as an integrative regulatory node that coordinates RNAPII remodeling, TFIIH recruitment, and PARP/BER crosstalk, while directing replication fork fate in a context-dependent manner, from protective stabilization to nuclease-mediated restart with a potential bias toward mutagenic break-induced replication.

The resulting outcome—genome-preserving repair versus error-tolerant rescue—is shaped by collision geometry, chromatin context, resource availability, and the checkpoint landscape. In tumors, where hypertranscription and epigenetic remodeling amplify TRCs, this balance becomes clinically decisive. A unifying view emerging from current evidence is that R-loops are not intrinsically deleterious; rather, their persistence at conflicted regions under high transcriptional flux favors fork failure. ERCC6-centered regulation operates as an adaptive regulatory axis: when RNAPII clearance and checkpoint control remain coordinated, genomic integrity is preserved; when this system is overloaded, cells resort to mutational tolerance mechanisms that promote adaptation under stress and drive genome evolution in proliferative disease contexts.

## Figures and Tables

**Table 1:** Major R-loop-processing factors and their established or proposed relationship with ERCC6/CSB.

Factor or Pathway	Principal Action on R-Loops	Relationship with ERCC6/CSB
RNase H1–RPA	RNase H1 hydrolyzes the RNA strand of the RNA:DNA hybrid. RPA binds the displaced ssDNA, promotes RNase H1 association with R-loops, and stimulates its catalytic activity [76].	A direct functional or recruitment relationship between CSB and RNase H1 has not been demonstrated. Although both proteins functionally intersect with RPA, whether CSB, RPA, and RNase H1 cooperate within a common R-loop-processing complex remains unknown [22,76].
FANCM, SMARCAL1, and ZRANB3	These branchpoint translocases can displace R-loops through ATP-dependent branchpoint translocation. FANCM deficiency causes R-loop accumulation, and depletion of FANCM, SMARCAL1, and ZRANB3 produces an additive R-loop accumulation and associated DNA damage [77].	No direct CSB relationship has been established for FANCM or ZRANB3. CSB directly competes with SMARCAL1 for RPA32 at stalled replication forks, and the functional consequences of this competition have been characterized particularly in BRCA2-deficient cells. However, this interaction has not been shown to coordinate R-loop displacement specifically [22].
SETX and AQR	SETX directly unwinds RNA hybrids and contributes to transcription termination and replication-fork progression. AQR is a spliceosome-associated RNA helicase whose depletion causes R-loop accumulation and R-loop-dependent DNA damage [28,78].	R-loops accumulating after SETX or AQR depletion can undergo CSB-, XPG-, and XPF-dependent processing into DSBs. This supports a sequential functional relationship rather than a demonstrated direct interaction between CSB and either SETX or AQR [28].
XPG and XPF–ERCC1	These structure-specific endonucleases mediate the conversion of persistent R-loops into DSBs under specific pathological conditions, including defective RNA processing or topoisomerase I inhibition [28].	XPG/XPF-dependent DSB formation from persistent R-loops requires CSB but not the global-genome NER factor XPC. However, direct recruitment of XPG or ERCC1–XPF by CSB to R-loops has not been demonstrated [28].

## Data Availability

No new data were created or analyzed in this study. Data sharing is not applicable to this article.

## Abbreviations

The following abbreviations are used in this manuscript:

ATR	Ataxia telangiectasia and Rad3-related kinase
BIR	Break-induced replication
CHK1	Checkpoint kinase 1
CS	Cockayne syndrome
CSA (ERCC8)	Cockayne syndrome group A protein
CSB (ERCC6)	Cockayne syndrome group B protein
DSB	Double-strand break
ecDNA	Extrachromosomal DNA
HR	Homologous recombination
HU	Hydroxyurea
MRE11	Meiotic recombination 11 homolog
NHEJ	Non-homologous end joining
PARP	Poly(ADP-ribose) polymerase
PCNA	Proliferating cell nuclear antigen
RAD52	RAD52 DNA repair protein
RIF1	Rap1-interacting factor 1
RNAPII	RNA polymerase II
ROS	Reactive oxygen species
RPA	Replication protein A
RS	Replication stress
ssDNA	Single-stranded DNA
TC-NER	Transcription-coupled nucleotide excision repair
TLS	Translesion synthesis
TOP2A	DNA topoisomerase II alpha
TRCs	Transcription–replication conflicts
UVSSA	UV-stimulated scaffold protein A
WEE1	WEE1 G2 checkpoint kinase
53BP1	p53-binding protein 1

## References

[1] Saxena S., Zou L. (2022). Hallmarks of DNA Replication Stress. Mol. Cell.

[2] Zeman M.K., Cimprich K.A. (2014). Causes and Consequences of Replication Stress. Nat. Cell Biol..

[3] Mahat D.B., Tippens N.D., Martin-Rufino J.D., Waterton S.K., Fu J., Blatt S.E., Sharp P.A. (2024). Single-Cell Nascent RNA Sequencing Unveils Coordinated Global Transcription. Nature.

[4] Pennycook B.R., Vesela E., Peripolli S., Singh T., Barr A.R., Bertoli C., De Bruin R.A.M. (2020). E2F-Dependent Transcription Determines Replication Capacity and S Phase Length. Nat. Commun..

[5] Armstrong C., Passanisi V.J., Ashraf H.M., Spencer S.L. (2023). Cyclin E/CDK2 and Feedback from Soluble Histone Protein Regulate the S Phase Burst of Histone Biosynthesis. Cell Rep..

[6] Mjelle R., Hegre S.A., Aas P.A., Slupphaug G., Drabløs F., Sætrom P., Krokan H.E. (2015). Cell Cycle Regulation of Human DNA Repair and Chromatin Remodeling Genes. DNA Repair.

[7] Bertoli C., Herlihy A.E., Pennycook B.R., Kriston-Vizi J., De Bruin R.A.M. (2016). Sustained E2F-Dependent Transcription Is a Key Mechanism to Prevent Replication-Stress-Induced DNA Damage. Cell Rep..

[8] Gaillard H., García-Muse T., Aguilera A. (2015). Replication Stress and Cancer. Nat. Rev. Cancer.

[9] García-Muse T., Aguilera A. (2016). Transcription–Replication Conflicts: How They Occur and How They Are Resolved. Nat. Rev. Mol. Cell Biol..

[10] Kotsantis P., Silva L.M., Irmscher S., Jones R.M., Folkes L., Gromak N., Petermann E. (2016). Increased Global Transcription Activity as a Mechanism of Replication Stress in Cancer. Nat. Commun..

[11] Helmrich A., Ballarino M., Tora L. (2011). Collisions between Replication and Transcription Complexes Cause Common Fragile Site Instability at the Longest Human Genes. Mol. Cell.

[12] Brison O., El-Hilali S., Azar D., Koundrioukoff S., Schmidt M., Nähse V., Jaszczyszyn Y., Lachages A.-M., Dutrillaux B., Thermes C. (2019). Transcription-Mediated Organization of the Replication Initiation Program across Large Genes Sets Common Fragile Sites Genome-Wide. Nat. Commun..

[13] Niehrs C., Luke B. (2020). Regulatory R-Loops as Facilitators of Gene Expression and Genome Stability. Nat. Rev. Mol. Cell Biol..

[14] Roy D., Yu K., Lieber M.R. (2008). Mechanism of R-Loop Formation at Immunoglobulin Class Switch Sequences. Mol. Cell. Biol..

[15] Crossley M.P., Bocek M., Cimprich K.A. (2019). R-Loops as Cellular Regulators and Genomic Threats. Mol. Cell.

[16] Petermann E., Lan L., Zou L. (2022). Sources, Resolution and Physiological Relevance of R-Loops and RNA–DNA Hybrids. Nat. Rev. Mol. Cell Biol..

[17] Rinaldi C., Pizzul P., Longhese M.P., Bonetti D. (2021). Sensing R-Loop-Associated DNA Damage to Safeguard Genome Stability. Front. Cell Dev. Biol..

[18] Kumar C., Remus D. (2024). Looping out of Control: R-Loops in Transcription-Replication Conflict. Chromosoma.

[19] Hamperl S., Bocek M.J., Saldivar J.C., Swigut T., Cimprich K.A. (2017). Transcription-Replication Conflict Orientation Modulates R-Loop Levels and Activates Distinct DNA Damage Responses. Cell.

[20] van der Weegen Y., Golan-Berman H., Mevissen T.E.T., Apelt K., González-Prieto R., Goedhart J., Heilbrun E.E., Vertegaal A.C.O., van den Heuvel D., Walter J.C. (2020). The Cooperative Action of CSB, CSA, and UVSSA Target TFIIH to DNA Damage-Stalled RNA Polymerase II. Nat. Commun..

[21] Duan M., Speer R.M., Ulibarri J., Liu K.J., Mao P. (2021). Transcription-Coupled Nucleotide Excision Repair: New Insights Revealed by Genomic Approaches. DNA Repair.

[22] Batenburg N.L., Sowa D.J., Walker J.R., Andres S.N., Zhu X.-D. (2024). CSB and SMARCAL1 Compete for RPA32 at Stalled Forks and Differentially Control the Fate of Stalled Forks in BRCA2-Deficient Cells. Nucleic Acids Res..

[23] Batenburg N.L., Mersaoui S.Y., Walker J.R., Coulombe Y., Hammond-Martel I., Wurtele H., Masson J.-Y., Zhu X.-D. (2021). Cockayne Syndrome Group B Protein Regulates Fork Restart, Fork Progression and MRE11-Dependent Fork Degradation in BRCA1/2-Deficient Cells. Nucleic Acids Res..

[24] Kokic G., Wagner F.R., Chernev A., Urlaub H., Cramer P. (2021). Structural Basis of Human Transcription–DNA Repair Coupling. Nature.

[25] Tan J., Duan M., Yadav T., Phoon L., Wang X., Zhang J.-M., Zou L., Lan L. (2020). An R-Loop-Initiated CSB–RAD52–POLD3 Pathway Suppresses ROS-Induced Telomeric DNA Breaks. Nucleic Acids Res..

[26] Bilkis R., Lake R.J., Fan H.-Y. (2025). ATP-Dependent Chromatin Remodeler CSB Couples DNA Repair Pathways to Transcription with Implications for Cockayne Syndrome and Cancer Therapy. Cells.

[27] Jeon J., Kang T.-H. (2025). Transcription-Coupled Repair and R-Loop Crosstalk in Genome Stability. Int. J. Mol. Sci..

[28] Sollier J., Stork C.T., García-Rubio M.L., Paulsen R.D., Aguilera A., Cimprich K.A. (2014). Transcription-Coupled Nucleotide Excision Repair Factors Promote R-Loop-Induced Genome Instability. Mol. Cell.

[29] Batenburg N.L., Walker J.R., Zhu X.-D. (2023). CSB Regulates Pathway Choice in Response to DNA Replication Stress Induced by Camptothecin. Int. J. Mol. Sci..

[30] Batenburg N.L., Walker J.R., Noordermeer S.M., Moatti N., Durocher D., Zhu X.-D. (2017). ATM and CDK2 Control Chromatin Remodeler CSB to Inhibit RIF1 in DSB Repair Pathway Choice. Nat. Commun..

[31] Pardo B., Aguilera A. (2012). Complex Chromosomal Rearrangements Mediated by Break-Induced Replication Involve Structure-Selective Endonucleases. PLoS Genet..

[32] Deem A., Keszthelyi A., Blackgrove T., Vayl A., Coffey B., Mathur R., Chabes A., Malkova A. (2011). Break-Induced Replication Is Highly Inaccurate. PLoS Biol..

[33] Spivak G. (2015). Nucleotide Excision Repair in Humans. DNA Repair.

[34] Girbig M., Misiaszek A.D., Müller C.W. (2022). Structural Insights into Nuclear Transcription by Eukaryotic DNA-Dependent RNA Polymerases. Nat. Rev. Mol. Cell Biol..

[35] Jacobs R.Q., Schneider D.A. (2024). Transcription Elongation Mechanisms of RNA Polymerases I, II, and III and Their Therapeutic Implications. J. Biol. Chem..

[36] Barba-Aliaga M., Alepuz P., Pérez-Ortín J.E. (2021). Eukaryotic RNA Polymerases: The Many Ways to Transcribe a Gene. Front. Mol. Biosci..

[37] Bradsher J., Auriol J., De Santis L.P., Iben S., Vonesch J.-L., Grummt I., Egly J.-M. (2002). CSB Is a Component of RNA Pol I Transcription. Mol. Cell.

[38] Yuan X., Feng W., Imhof A., Grummt I., Zhou Y. (2007). Activation of RNA Polymerase I Transcription by Cockayne Syndrome Group B Protein and Histone Methyltransferase G9a. Mol. Cell.

[39] Cho I., Tsai P.-F., Lake R.J., Basheer A., Fan H.-Y. (2013). ATP-Dependent Chromatin Remodeling by Cockayne Syndrome Protein B and NAP1-Like Histone Chaperones Is Required for Efficient Transcription-Coupled DNA Repair. PLoS Genet..

[40] Yu A., Fan H.-Y., Liao D., Bailey A.D., Weiner A.M. (2000). Activation of P53 or Loss of the Cockayne Syndrome Group B Repair Protein Causes Metaphase Fragility of Human U1, U2, and 5S Genes. Mol. Cell.

[41] Lalonde M., Trauner M., Werner M., Hamperl S. (2021). Consequences and Resolution of Transcription–Replication Conflicts. Life.

[42] Browning K.R., Merrikh H. (2024). Replication–Transcription Conflicts: A Perpetual War on the Chromosome. Annu. Rev. Biochem..

[43] Bowry A., Kelly R.D.W., Petermann E. (2021). Hypertranscription and Replication Stress in Cancer. Trends Cancer.

[44] Chen Y.-H., Keegan S., Kahli M., Tonzi P., Fenyö D., Huang T.T., Smith D.J. (2019). Transcription Shapes DNA Replication Initiation and Termination in Human Cells. Nat. Struct. Mol. Biol..

[45] Musvi S.S., Moiseeva T.N. (2025). From Cooperation to Conflicts—A Complicated Relationship between Transcription and Replication. DNA Repair.

[46] Ge X.Q., Jackson D.A., Blow J.J. (2007). Dormant Origins Licensed by Excess Mcm2–7 Are Required for Human Cells to Survive Replicative Stress. Genes Dev..

[47] Sedlackova H., Rask M.-B., Gupta R., Choudhary C., Somyajit K., Lukas J. (2020). Equilibrium between Nascent and Parental MCM Proteins Protects Replicating Genomes. Nature.

[48] McIntosh D., Blow J.J. (2012). Dormant Origins, the Licensing Checkpoint, and the Response to Replicative Stresses. Cold Spring Harb. Perspect. Biol..

[49] Ibarra A., Schwob E., Méndez J. (2008). Excess MCM Proteins Protect Human Cells from Replicative Stress by Licensing Backup Origins of Replication. Proc. Natl. Acad. Sci. USA.

[50] Hsu C.-L., Chong S.Y., Lin C.-Y., Kao C.-F. (2021). Histone Dynamics during DNA Replication Stress. J. Biomed. Sci..

[51] Jaspers N.G.J., Raams A., Kelner M.J., Ng J.M.Y., Yamashita Y.M., Takeda S., McMorris T.C., Hoeijmakers J.H.J. (2002). Anti-Tumour Compounds Illudin S and Irofulven Induce DNA Lesions Ignored by Global Repair and Exclusively Processed by Transcription- and Replication-Coupled Repair Pathways. DNA Repair.

[52] Lans H., Hoeijmakers J.H.J., Vermeulen W., Marteijn J.A. (2019). The DNA Damage Response to Transcription Stress. Nat. Rev. Mol. Cell Biol..

[53] Casas-Delucchi C.S., Daza-Martin M., Williams S.L., Coster G. (2022). The Mechanism of Replication Stalling and Recovery within Repetitive DNA. Nat. Commun..

[54] Williams S.L., Casas-Delucchi C.S., Raguseo F., Guneri D., Li Y., Minamino M., Fletcher E.E., Yeeles J.T., Keyser U.F., Waller Z.A. (2023). Replication-induced DNA Secondary Structures Drive Fork Uncoupling and Breakage. EMBO J..

[55] Nikolov I., Taddei A. (2016). Linking Replication Stress with Heterochromatin Formation. Chromosoma.

[56] Nathanailidou P., Dhakshnamoorthy J., Xiao H., Zofall M., Holla S., O’Neill M., Andresson T., Wheeler D., Grewal S.I.S. (2024). Specialized Replication of Heterochromatin Domains Ensures Self-Templated Chromatin Assembly and Epigenetic Inheritance. Proc. Natl. Acad. Sci. USA.

[57] Stengel K.R., Hiebert S.W. (2015). Class I HDACs Affect DNA Replication, Repair, and Chromatin Structure: Implications for Cancer Therapy. Antioxid. Redox Signal..

[58] Wells C.E., Bhaskara S., Stengel K.R., Zhao Y., Sirbu B., Chagot B., Cortez D., Khabele D., Chazin W.J., Cooper A. (2013). Inhibition of Histone Deacetylase 3 Causes Replication Stress in Cutaneous T Cell Lymphoma. PLoS ONE.

[59] Schvartzman J.-M., Forsyth G., Walch H., Chatila W., Taglialatela A., Lee B.J., Zhu X., Gershik S., Cimino F.V., Santella A. (2023). Oncogenic IDH Mutations Increase Heterochromatin-Related Replication Stress without Impacting Homologous Recombination. Mol. Cell.

[60] Marteijn J.A., Lans H., Vermeulen W., Hoeijmakers J.H.J. (2014). Understanding Nucleotide Excision Repair and Its Roles in Cancer and Ageing. Nat. Rev. Mol. Cell Biol..

[61] Dinant C., Ampatziadis-Michailidis G., Lans H., Tresini M., Lagarou A., Grosbart M., Theil A.F., van Cappellen W.A., Kimura H., Bartek J. (2013). Enhanced Chromatin Dynamics by FACT Promotes Transcriptional Restart after UV-Induced DNA Damage. Mol. Cell.

[62] Werner M., Trauner M., Schauer T., Ummethum H., Márquez-Gómez E., Lalonde M., Lee C.S.K., Tsirkas I., Sajid A., Murriello A.C. (2025). Transcription-Replication Conflicts Drive R-Loop-Dependent Nucleosome Eviction and Require DOT1L Activity for Transcription Recovery. Nucleic Acids Res..

[63] Oksenych V., Zhovmer A., Ziani S., Mari P.-O., Eberova J., Nardo T., Stefanini M., Giglia-Mari G., Egly J.-M., Coin F. (2013). Histone Methyltransferase DOT1L Drives Recovery of Gene Expression after a Genotoxic Attack. PLoS Genet..

[64] Tian M., Alt F.W. (2000). Transcription-Induced Cleavage of Immunoglobulin Switch Regions by Nucleotide Excision Repair Nucleases in Vitro. J. Biol. Chem..

[65] Ginno P.A., Lott P.L., Christensen H.C., Korf I., Chédin F. (2012). R-Loop Formation Is a Distinctive Characteristic of Unmethylated Human CpG Island Promoters. Mol. Cell.

[66] Skourti-Stathaki K., Proudfoot N.J., Gromak N. (2011). Human Senataxin Resolves RNA/DNA Hybrids Formed at Transcriptional Pause Sites to Promote Xrn2-Dependent Termination. Mol. Cell.

[67] Sanz L.A., Hartono S.R., Lim Y.W., Steyaert S., Rajpurkar A., Ginno P.A., Xu X., Chédin F. (2016). Prevalent, Dynamic, and Conserved R-Loop Structures Associate with Specific Epigenomic Signatures in Mammals. Mol. Cell.

[68] Domínguez-Sánchez M.S., Barroso S., Gómez-González B., Luna R., Aguilera A. (2011). Genome Instability and Transcription Elongation Impairment in Human Cells Depleted of THO/TREX. PLoS Genet..

[69] Teng Y., Yu Y., Li S., Huang Y., Xu D., Tao X., Fan Y. (2021). Ultraviolet Radiation and Basal Cell Carcinoma: An Environmental Perspective. Front. Public Health.

[70] Gan W., Guan Z., Liu J., Gui T., Shen K., Manley J.L., Li X. (2011). R-Loop-Mediated Genomic Instability Is Caused by Impairment of Replication Fork Progression. Genes Dev..

[71] Llerena Schiffmacher D.A., Pai Y.J., Pines A., Vermeulen W. (2025). Transcription-coupled Repair: Tangled up in Convoluted Repair. FEBS J..

[72] Zhang X., Xu J., Hu J., Zhang S., Hao Y., Zhang D., Qian H., Wang D., Fu X.-D. (2024). Cockayne Syndrome Linked to Elevated R-Loops Induced by Stalled RNA Polymerase II during Transcription Elongation. Nat. Commun..

[73] Xu J., Wang W., Xu L., Chen J.-Y., Chong J., Oh J., Leschziner A.E., Fu X.-D., Wang D. (2020). Cockayne Syndrome B Protein Acts as an ATP-Dependent Processivity Factor That Helps RNA Polymerase II Overcome Nucleosome Barriers. Proc. Natl. Acad. Sci. USA.

[74] Teng Y., Yadav T., Duan M., Tan J., Xiang Y., Gao B., Xu J., Liang Z., Liu Y., Nakajima S. (2018). ROS-Induced R Loops Trigger a Transcription-Coupled but BRCA1/2-Independent Homologous Recombination Pathway through CSB. Nat. Commun..

[75] Jalan M., Sharma A., Pei X., Weinhold N., Buechelmaier E.S., Zhu Y., Ahmed-Seghir S., Ratnakumar A., Di Bona M., McDermott N. (2024). RAD52 Resolves Transcription-Replication Conflicts to Mitigate R-Loop Induced Genome Instability. Nat. Commun..

[76] Nguyen H.D., Yadav T., Giri S., Saez B., Graubert T.A., Zou L. (2017). Functions of Replication Protein A as a Sensor of R Loops and a Regulator of RNaseH1. Mol. Cell.

[77] Hodson C., Van Twest S., Dylewska M., O’Rourke J.J., Tan W., Murphy V.J., Walia M., Abbouche L., Nieminuszczy J., Dunn E. (2022). Branchpoint Translocation by Fork Remodelers as a General Mechanism of R-Loop Removal. Cell Rep..

[78] Hasanova Z., Klapstova V., Porrua O., Stefl R., Sebesta M. (2023). Human Senataxin Is a Bona Fide R-Loop Resolving Enzyme and Transcription Termination Factor. Nucleic Acids Res..

[79] Lam F.C., Kong Y.W., Huang Q., Vu Han T.-L., Maffa A.D., Kasper E.M., Yaffe M.B. (2020). BRD4 Prevents the Accumulation of R-Loops and Protects against Transcription–Replication Collision Events and DNA Damage. Nat. Commun..

[80] Zambrano E., Fierro C., Morales F., Manterola M., Marin A., Armisen R., Marcelain K. (2026). Transcription-Coupled Repair Promotes the Retention of Mutations in Coding Regions During Replication Stress. Int. J. Mol. Sci..

[81] Spivak G. (2016). Transcription-Coupled Repair: An Update. Arch. Toxicol..

[82] Walker J.R., Zhu X.-D. (2022). Role of Cockayne Syndrome Group B Protein in Replication Stress: Implications for Cancer Therapy. Int. J. Mol. Sci..

[83] Tiwari V., Baptiste B.A., Okur M.N., Bohr V.A. (2021). Current and Emerging Roles of Cockayne Syndrome Group B (CSB) Protein. Nucleic Acids Res..

[84] Van Der Weegen Y., De Lint K., Van Den Heuvel D., Nakazawa Y., Mevissen T.E.T., Van Schie J.J.M., San Martin Alonso M., Boer D.E.C., González-Prieto R., Narayanan I.V. (2021). ELOF1 Is a Transcription-Coupled DNA Repair Factor That Directs RNA Polymerase II Ubiquitylation. Nat. Cell Biol..

[85] Ramadhin A.R., Lee S.-H., Zhou D., Salmazo A., Gonzalo-Hansen C., Van Sluis M., Blom C.M.A., Janssens R.C., Raams A., Dekkers D. (2024). STK19 Drives Transcription-Coupled Repair by Stimulating Repair Complex Stability, RNA Pol II Ubiquitylation, and TFIIH Recruitment. Mol. Cell.

[86] Van Den Heuvel D., Rodríguez-Martínez M., Van Der Meer P.J., Nieto Moreno N., Park J., Kim H.-S., Van Schie J.J.M., Wondergem A.P., D’Souza A., Yakoub G. (2024). STK19 Facilitates the Clearance of Lesion-Stalled RNAPII during Transcription-Coupled DNA Repair. Cell.

[87] Thorslund T., Von Kobbe C., Harrigan J.A., Indig F.E., Christiansen M., Stevnsner T., Bohr V.A. (2005). Cooperation of the Cockayne Syndrome Group B Protein and Poly(ADP-Ribose) Polymerase 1 in the Response to Oxidative Stress. Mol. Cell. Biol..

[88] Boetefuer E.L., Lake R.J., Dreval K., Fan H.-Y. (2018). Poly(ADP-Ribose) Polymerase 1 (PARP1) Promotes Oxidative Stress–Induced Association of Cockayne Syndrome Group B Protein with Chromatin. J. Biol. Chem..

[89] Bilkis R., Lake R.J., Cooper K.L., Tomkinson A., Fan H.-Y. (2023). The CSB Chromatin Remodeler Regulates PARP1- and PARP2-Mediated Single-Strand Break Repair at Actively Transcribed DNA Regions. Nucleic Acids Res..

[90] Sarmini L., Meabed M., Emmanouil E., Atsaves G., Robeska E., Karwowski B.T., Campalans A., Gimisis T., Khobta A. (2023). Requirement of Transcription-Coupled Nucleotide Excision Repair for the Removal of a Specific Type of Oxidatively Induced DNA Damage. Nucleic Acids Res..

[91] Zhu Y., Zhang X., Gao M., Huang Y., Tan Y., Parnas A., Wu S., Zhan D., Adar S., Hu J. (2024). Coordination of Transcription-Coupled Repair and Repair-Independent Release of Lesion-Stalled RNA Polymerase II. Nat. Commun..

[92] Gonzalo-Hansen C., Steurer B., Janssens R.C., Zhou D., van Sluis M., Lans H., Marteijn J.A. (2024). Differential Processing of RNA Polymerase II at DNA Damage Correlates with Transcription-Coupled Repair Syndrome Severity. Nucleic Acids Res..

[93] Luo Y., Li J., Li X., Lin H., Mao Z., Xu Z., Li S., Nie C., Zhou X.A., Liao J. (2024). The ARK2N–CK2 Complex Initiates Transcription-Coupled Repair through Enhancing the Interaction of CSB with Lesion-Stalled RNAPII. Proc. Natl. Acad. Sci. USA.

[94] Van Schie J.J.M., Brussee S.J., Luijsterburg M.S. (2025). Evidence That ARK2N Is Not a Core Factor in Transcription-Coupled DNA Repair. Proc. Natl. Acad. Sci. USA.

[95] Liano D., Chowdhury S., Di Antonio M. (2021). Cockayne Syndrome B Protein Selectively Resolves and Interact with Intermolecular DNA G-Quadruplex Structures. J. Am. Chem. Soc..

[96] Spyropoulou Z., Papaspyropoulos A., Lagopati N., Myrianthopoulos V., Georgakilas A.G., Fousteri M., Kotsinas A., Gorgoulis V.G. (2021). Cockayne Syndrome Group B (CSB): The Regulatory Framework Governing the Multifunctional Protein and Its Plausible Role in Cancer. Cells.

[97] Konovalov D., Umerenkov D., Herbert A., Poptsova M. (2025). GQ-DNABERT Reveals GQ Proximal Enhancer–Promoter Interactions Associated with Tissue-Specific Transcription. Nucleic Acids Res..

[98] Antariksa N.F., Di Antonio M. (2024). The Emerging Roles of Multimolecular G-Quadruplexes in Transcriptional Regulation and Chromatin Organization. Acc. Chem. Res..

[99] Hanada K., Budzowska M., Davies S.L., Van Drunen E., Onizawa H., Beverloo H.B., Maas A., Essers J., Hickson I.D., Kanaar R. (2007). The Structure-Specific Endonuclease Mus81 Contributes to Replication Restart by Generating Double-Strand DNA Breaks. Nat. Struct. Mol. Biol..

[100] Regairaz M., Zhang Y.-W., Fu H., Agama K.K., Tata N., Agrawal S., Aladjem M.I., Pommier Y. (2011). Mus81-Mediated DNA Cleavage Resolves Replication Forks Stalled by Topoisomerase I–DNA Complexes. J. Cell Biol..

[101] Patil M., Pabla N., Dong Z. (2013). Checkpoint Kinase 1 in DNA Damage Response and Cell Cycle Regulation. Cell. Mol. Life Sci..

[102] Matsuno Y., Hyodo M., Suzuki M., Tanaka Y., Horikoshi Y., Murakami Y., Torigoe H., Mano H., Tashiro S., Yoshioka K.-I. (2021). Replication-Stress-Associated DSBs Induced by Ionizing Radiation Risk Genomic Destabilization and Associated Clonal Evolution. iScience.

[103] Wilhelm T., Ragu S., Magdalou I., Machon C., Dardillac E., Técher H., Guitton J., Debatisse M., Lopez B.S. (2016). Slow Replication Fork Velocity of Homologous Recombination-Defective Cells Results from Endogenous Oxidative Stress. PLoS Genet..

[104] Cui S., Walker J.R., Batenburg N.L., Zhu X.-D. (2022). Cockayne Syndrome Group B Protein Uses Its DNA Translocase Activity to Promote Mitotic DNA Synthesis. DNA Repair.

[105] Tsaalbi-Shtylik A., Moser J., Mullenders L.H.F., Jansen J.G., de Wind N. (2014). Persistently Stalled Replication Forks Inhibit Nucleotide Excision Repair in Trans by Sequestering Replication Protein A. Nucleic Acids Res..

[106] Stern H.R., Sefcikova J., Chaparro V.E., Beuning P.J. (2019). Mammalian DNA Polymerase Kappa Activity and Specificity. Molecules.

[107] Layer J.V., Debaize L., Van Scoyk A., House N.C., Brown A.J., Liu Y., Stevenson K.E., Hemann M., Roberts S.A., Price B.D. (2020). Polymerase δ Promotes Chromosomal Rearrangements and Imprecise Double-Strand Break Repair. Proc. Natl. Acad. Sci. USA.

[108] Vaziri C., Rogozin I.B., Gu Q., Wu D., Day T.A. (2021). Unravelling Roles of Error-Prone DNA Polymerases in Shaping Cancer Genomes. Oncogene.

[109] Nieto Moreno N., Olthof A.M., Svejstrup J.Q. (2023). Transcription-Coupled Nucleotide Excision Repair and the Transcriptional Response to UV-Induced DNA Damage. Annu. Rev. Biochem..

[110] Scully R., Xie A. (2013). Double Strand Break Repair Functions of Histone H2AX. Mutat. Res. Mol. Mech. Mutagen..

[111] Feng Y.-L., Xiang J.-F., Liu S.-C., Guo T., Yan G.-F., Feng Y., Kong N., Li H.-D., Huang Y., Lin H. (2017). H2AX Facilitates Classical Non-Homologous End Joining at the Expense of Limited Nucleotide Loss at Repair Junctions. Nucleic Acids Res..

[112] Rother M.B., Pellegrino S., Smith R., Gatti M., Meisenberg C., Wiegant W.W., Luijsterburg M.S., Imhof R., Downs J.A., Vertegaal A.C.O. (2020). CHD7 and 53BP1 Regulate Distinct Pathways for the Re-Ligation of DNA Double-Strand Breaks. Nat. Commun..

[113] Sishc B., Davis A. (2017). The Role of the Core Non-Homologous End Joining Factors in Carcinogenesis and Cancer. Cancers.

[114] Costantino L., Sotiriou S.K., Rantala J.K., Magin S., Mladenov E., Helleday T., Haber J.E., Iliakis G., Kallioniemi O.P., Halazonetis T.D. (2014). Break-Induced Replication Repair of Damaged Forks Induces Genomic Duplications in Human Cells. Science.

[115] Aird K.M., Zhang G., Li H., Tu Z., Bitler B.G., Garipov A., Wu H., Wei Z., Wagner S.N., Herlyn M. (2013). Suppression of Nucleotide Metabolism Underlies the Establishment and Maintenance of Oncogene-Induced Senescence. Cell Rep..

[116] Mannava S., Moparthy K.C., Wheeler L.J., Natarajan V., Zucker S.N., Fink E.E., Im M., Flanagan S., Burhans W.C., Zeitouni N.C. (2013). Depletion of Deoxyribonucleotide Pools Is an Endogenous Source of DNA Damage in Cells Undergoing Oncogene-Induced Senescence. Am. J. Pathol..

[117] Bester A.C., Roniger M., Oren Y.S., Im M.M., Sarni D., Chaoat M., Bensimon A., Zamir G., Shewach D.S., Kerem B. (2011). Nucleotide Deficiency Promotes Genomic Instability in Early Stages of Cancer Development. Cell.

[118] Martin H.A., Pedraza-Reyes M., Yasbin R.E., Robleto E.A. (2011). Transcriptional De-Repression and Mfd Are Mutagenic in Stressed Bacillus Subtilis Cells. J. Mol. Microbiol. Biotechnol..

[119] Million-Weaver S., Samadpour A.N., Moreno-Habel D.A., Nugent P., Brittnacher M.J., Weiss E., Hayden H.S., Miller S.I., Liachko I., Merrikh H. (2015). An Underlying Mechanism for the Increased Mutagenesis of Lagging-Strand Genes in *Bacillus subtilis*. Proc. Natl. Acad. Sci. USA.

[120] Han J., Sahin O., Barton Y.-W., Zhang Q. (2008). Key Role of Mfd in the Development of Fluoroquinolone Resistance in Campylobacter Jejuni. PLoS Pathog..

[121] Ragheb M.N., Thomason M.K., Hsu C., Nugent P., Gage J., Samadpour A.N., Kariisa A., Merrikh C.N., Miller S.I., Sherman D.R. (2019). Inhibiting the Evolution of Antibiotic Resistance. Mol. Cell.

[122] Gu L., Hickey R.J., Malkas L.H. (2023). Therapeutic Targeting of DNA Replication Stress in Cancer. Genes.

[123] Da Costa A.A.B.A., Chowdhury D., Shapiro G.I., D’Andrea A.D., Konstantinopoulos P.A. (2023). Targeting Replication Stress in Cancer Therapy. Nat. Rev. Drug Discov..

[124] Nickoloff J.A., Jaiswal A.S., Sharma N., Williamson E.A., Tran M.T., Arris D., Yang M., Hromas R. (2023). Cellular Responses to Widespread DNA Replication Stress. Int. J. Mol. Sci..

[125] Hills S.A., Diffley J.F.X. (2014). DNA Replication and Oncogene-Induced Replicative Stress. Curr. Biol..

[126] Macheret M., Halazonetis T.D. (2015). DNA Replication Stress as a Hallmark of Cancer. Annu. Rev. Pathol. Mech. Dis..

[127] Caputo M., Frontini M., Velez-Cruz R., Nicolai S., Prantera G., Proietti-De-Santis L. (2013). The CSB Repair Factor Is Overexpressed in Cancer Cells, Increases Apoptotic Resistance, and Promotes Tumor Growth. DNA Repair.

[128] Proietti-De-Santis L., Balzerano A., Prantera G. (2018). CSB: An Emerging Actionable Target for Cancer Therapy. Trends Cancer.

[129] Yadav S., Anbalagan M., Baddoo M., Chellamuthu V.K., Mukhopadhyay S., Woods C., Jiang W., Moroz K., Flemington E.K., Makridakis N. (2020). Somatic Mutations in the DNA Repairome in Prostate Cancers in African Americans and Caucasians. Oncogene.

[130] Ogundepo O., De Benedetti A. (2026). Studies on the Functionality of the TC-NER ERCC6-M1097V Protein Variant Frequently Found in Louisiana Patients with PCa upon UV Damage. Front. Oncol..

[131] Kovalenko T.F., Abdurazakov A., Antipova N.V., Shakhparonov M.I., Pavlyukov M.S. (2025). R-Loops as a Trigger for Intra- and Extrachromosomal DNA Amplification in Cancer. Front. Cell Dev. Biol..

[132] Tang J., Weiser N.E., Wang G., Chowdhry S., Curtis E.J., Zhao Y., Wong I.T.-L., Marinov G.K., Li R., Hanoian P. (2024). Enhancing Transcription–Replication Conflict Targets ecDNA-Positive Cancers. Nature.

[133] Thangaretnam K., Islam M.O., Lv J., El-Rifai A., Perloff A., Soutto H.L., Peng D., Chen Z. (2025). WEE1 Inhibition in Cancer Therapy: Mechanisms, Synergies, Preclinical Insights, and Clinical Trials. Crit. Rev. Oncol. Hematol..

[134] Xu R., Zhu S., Zhang W., Xu H., Tu C., Wang H., Wang L., He N., Liu T., Guo X. (2025). A Dual Approach with Organoid and CRISPR Screening Reveals ERCC6 as a Determinant of Cisplatin Resistance in Osteosarcoma. Adv. Sci..

[135] Musiałek M.W., Rybaczek D. (2021). Hydroxyurea—The Good, the Bad and the Ugly. Genes.

[136] Navarro-Jiménez M., González B., Mulet N., Hierro C., Alonso S. (2025). KRAS-Targeted Therapies in Colorectal Cancer: A Systematic Analysis of Mutations, Inhibitors, and Clinical Trials. npj Precis. Oncol..

[137] Saeed H., Leibowitz B.J., Zhang L., Yu J. (2023). Targeting Myc-Driven Stress Addiction in Colorectal Cancer. Drug Resist. Updates.

[138] Wallbillich N.J., Lu H. (2023). Role of C-Myc in Lung Cancer: Progress, Challenges, and Prospects. Chin. Med. J. Pulm. Crit. Care Med..

[139] Karimi N., Moghaddam S.J. (2023). KRAS-Mutant Lung Cancer: Targeting Molecular and Immunologic Pathways, Therapeutic Advantages and Restrictions. Cells.

[140] Jiang Y., Mai G., Zhao X., Tang M., Yang P., Cheng Q., Tian H., Niu Z., Wang X., Wang J. (2025). Molecular Characterization and Prognostic Implications of KRAS Mutations in Pancreatic Cancer Patients: Insights from Multi-Cohort Analysis. npj Precis. Oncol..

[141] Curti L., Campaner S. (2021). MYC-Induced Replicative Stress: A Double-Edged Sword for Cancer Development and Treatment. Int. J. Mol. Sci..

[142] Zhang Y. (2025). Exploiting Replication Stress for Synthetic Lethality in MYC-Driven Cancers. Am. J. Cancer Res..

[143] Técher H., Kemiha S., Aobuli X., Kolinjivadi A.M. (2024). Oncogenic RAS in Cancers from the DNA Replication Stress and Senescence Perspective. Cancers.

[144] Smith S.J., Meng F., Lingeman R.G., Li C.M., Li M., Boneh G., Seppälä T.T., Phan T., Li H., Burkhart R.A. (2025). Therapeutic Targeting of Oncogene-Induced Transcription-Replication Conflicts in Pancreatic Ductal Adenocarcinoma. Gastroenterology.

[145] Ito S.S., Nakagawa Y., Matsubayashi M., Sakaguchi Y.M., Kobashigawa S., Matsui T.K., Nanaura H., Nakanishi M., Kitayoshi F., Kikuchi S. (2020). Inhibition of the ATR Kinase Enhances 5-FU Sensitivity Independently of Nonhomologous End-Joining and Homologous Recombination Repair Pathways. J. Biol. Chem..

[146] Colicchia V., Petroni M., Guarguaglini G., Sardina F., Sahún-Roncero M., Carbonari M., Ricci B., Heil C., Capalbo C., Belardinilli F. (2017). PARP Inhibitors Enhance Replication Stress and Cause Mitotic Catastrophe in MYCN-Dependent Neuroblastoma. Oncogene.

[147] Parsels L.A., Karnak D., Parsels J.D., Zhang Q., Vélez-Padilla J., Reichert Z.R., Wahl D.R., Maybaum J., O’Connor M.J., Lawrence T.S. (2018). PARP1 Trapping and DNA Replication Stress Enhance Radiosensitization with Combined WEE1 and PARP Inhibitors. Mol. Cancer Res..

[148] Smith H.L., Willmore E., Prendergast L., Curtin N.J. (2024). ATR, CHK1 and WEE1 Inhibitors Cause Homologous Recombination Repair Deficiency to Induce Synthetic Lethality with PARP Inhibitors. Br. J. Cancer.

[149] Kumar N.A., Manasa P., Chandrasekaran J. (2025). Revisiting CHK1 and WEE1 Kinases as Therapeutic Targets in Cancer. Biochim. Biophys. Acta BBA-Rev. Cancer.

[150] Yano K., Shiotani B. (2023). Emerging Strategies for Cancer Therapy by ATR Inhibitors. Cancer Sci..

[151] Purshouse K., Friman E.T., Boyle S., Dewari P.S., Grant V., Hamdan A., Morrison G.M., Brennan P.M., Beentjes S.V., Pollard S.M. (2022). Oncogene Expression from Extrachromosomal DNA Is Driven by Copy Number Amplification and Does Not Require Spatial Clustering in Glioblastoma Stem Cells. eLife.

[152] Stöber M.C., Chamorro González R., Brückner L., Conrad T., Wittstruck N., Szymansky A., Eggert A., Schulte J.H., Koche R.P., Henssen A.G. (2024). Intercellular Extrachromosomal DNA Copy-Number Heterogeneity Drives Neuroblastoma Cell State Diversity. Cell Rep..

[153] Koppenhafer S.L., Geary E.L., Thomas M.V., Croushore E.E., Zimmerman J.A.O., Gedminas J.M., Quelle D.E., Dodd R.D., Gordon D.J. (2025). Histone Deacetylase Inhibitors Target DNA Replication Regulators and Replication Stress in Ewing Sarcoma Cells. Cancer Res. Commun..

[154] Li Y., Seto E. (2016). HDACs and HDAC Inhibitors in Cancer Development and Therapy. Cold Spring Harb. Perspect. Med..

[155] Byrd A.K. (2012). Superfamily 2 Helicases. Front. Biosci..

[156] Wang L., Limbo O., Fei J., Chen L., Kim B., Luo J., Chong J., Conaway R.C., Conaway J.W., Ranish J.A. (2014). Regulation of the Rhp26^ERCC6/CSB^ Chromatin Remodeler by a Novel Conserved Leucine Latch Motif. Proc. Natl. Acad. Sci. USA.

[157] Caputo M., Balzerano A., Arisi I., D’Onofrio M., Brandi R., Bongiorni S., Brancorsini S., Frontini M., Proietti-De-Santis L. (2017). CSB Ablation Induced Apoptosis Is Mediated by Increased Endoplasmic Reticulum Stress Response. PLoS ONE.

[158] Nicolai S., Filippi S., Caputo M., Cipak L., Gregan J., Ammerer G., Frontini M., Willems D., Prantera G., Balajee A.S. (2015). Identification of Novel Proteins Co-Purifying with Cockayne Syndrome Group B (CSB) Reveals Potential Roles for CSB in RNA Metabolism and Chromatin Dynamics. PLoS ONE.

[159] Haubold M.K., Aquino J.N.P., Rubin S.R., Jones I.K., Larsen C.I.S., Pham E., Majumder K. (2023). Genomes of the Autonomous Parvovirus Minute Virus of Mice Induce Replication Stress through RPA Exhaustion. PLoS Pathog..

[160] Ibler A.E.M., ElGhazaly M., Naylor K.L., Bulgakova N.A., El-Khamisy S.F., Humphreys D. (2019). Typhoid Toxin Exhausts the RPA Response to DNA Replication Stress Driving Senescence and Salmonella Infection. Nat. Commun..

[161] Moody C.A. (2019). Impact of Replication Stress in Human Papillomavirus Pathogenesis. J. Virol..

[162] Sakakibara N., Mitra R., McBride A.A. (2011). The Papillomavirus E1 Helicase Activates a Cellular DNA Damage Response in Viral Replication Foci. J. Virol..

[163] Saladino N., Salamango D.J. (2024). Wielding a Double-Edged Sword: Viruses Exploit Host DNA Repair Systems to Facilitate Replication While Bypassing Immune Activation. Front. Virol..

[164] Li T., Chen Z.J. (2018). The cGAS–cGAMP–STING Pathway Connects DNA Damage to Inflammation, Senescence, and Cancer. J. Exp. Med..

[165] Newman L.E., Weiser Novak S., Rojas G.R., Tadepalle N., Schiavon C.R., Grotjahn D.A., Towers C.G., Tremblay M.-È., Donnelly M.P., Ghosh S. (2024). Mitochondrial DNA Replication Stress Triggers a Pro-Inflammatory Endosomal Pathway of Nucleoid Disposal. Nat. Cell Biol..

[166] Zannini L., Cardano M., Liberi G., Buscemi G. (2024). R-Loops and Impaired Autophagy Trigger cGAS-Dependent Inflammation via Micronuclei Formation in Senataxin-Deficient Cells. Cell. Mol. Life Sci..

[167] Cancado De Faria R., Silva L.N.D., Teodoro-Castro B., McCommis K.S., Shashkova E.V., Gonzalo S. (2025). A Noncanonical cGAS–STING Pathway Drives Cellular and Organismal Aging. Proc. Natl. Acad. Sci. USA.

